# New York State Climate Impacts Assessment Chapter 08: Society and Economy

**DOI:** 10.1111/nyas.15199

**Published:** 2024-12-09

**Authors:** Luis Aguirre‐Torres, Robin Leichenko, Mary Austerman, Deborah Balk, Hallie E. Bond, Riobart E. Breen, David Burgy, Cassandra John, Franchelle Parker, Kenneth Schlather, Amanda Stevens

**Affiliations:** ^1^ New York State Energy Research and Development Authority Albany New York USA; ^2^ Department of Geography Rutgers University New Brunswick New Jersey USA; ^3^ New York Sea Grant Newark New York USA; ^4^ CUNY Institute for Demographic Research and Marxe School of Public and International Affairs, Baruch College The City University of New York New York New York USA; ^5^ Independent Historian Long Lake New York USA; ^6^ New York State Department of Environmental Conservation Albany New York USA; ^7^ Independent Consultant New York New York USA; ^8^ Sif Capital Advisors Saratoga Springs New York USA; ^9^ Open Buffalo Buffalo New York USA; ^10^ Independent Consultant Ithaca New York USA

**Keywords:** adaptation, climate change, culture, economy, impacts, New York State, resilience, tourism, vulnerability

## Abstract

Climate change is affecting or will affect the lives of every resident of New York State. This chapter examines the impacts of climate change on five critical areas in the state: populations and migration, the economy, education, culture, and government. The chapter highlights differential vulnerabilities among the state's regions, populations, workers, and businesses, paying particular attention to issues of equity and environmental justice.

## 1 TECHNICAL WORKGROUP KEY FINDINGS

Climate change is affecting or will affect the lives of every resident of New York State. This chapter examines the impacts of climate change on five critical areas in the state: populations and migration, the economy, education, culture, and government. The chapter highlights differential vulnerabilities among the state's regions, populations, workers, and businesses, paying particular attention to issues of equity and environmental justice.

**Key Finding 1: Climate change is already affecting and will increasingly affect nearly every dimension of New York State's economy**. Climate hazards such as rising temperatures and more extreme weather events will directly or indirectly affect nearly all economic sectors in the state, from natural resource−based industries to manufacturing, retail, and financial services. To benefit all New Yorkers, climate solutions will need to consider those who are disproportionately burdened by economic disruption, such as small businesses; fiscally constrained, small, and rural municipalities and cultural institutions; frontline workers; and essential public servants including police, firefighters, and teachers.

**Key Finding 2: New York State's older residents are more vulnerable and less able to adapt to heat, flooding, and other climate hazards**. New York State's population is aging, especially in rural areas where the portion of the population over 65 is growing at a much faster rate than in urban areas. Addressing the unique vulnerabilities of older New Yorkers will require special attention to emergency preparedness and disaster relief planning.

**Key Finding 3: Climate change vulnerabilities intersect with and are exacerbated by underlying and systemic social/economic stressors and fragilities**. Low‐income populations in New York State, notably those who are Indigenous, people of color, immigrants, unhoused, or live in rural areas, are more vulnerable due to existing forms of disadvantage and marginalization, including legacies of displacement, racial and ethnic discrimination, limited access to resources, and higher exposure to environmental pollutants. Recognition of and attention to the needs of historically underserved and overburdened populations are critical elements of equitable and effective resilience planning.

**Key Finding 4: Climate change threatens Indigenous communities in New York State and their natural resource−based cultural heritages and traditions**. Loss of heritage sites due to sea level rise, reductions in opportunities for traditional livelihood practices such as ice fishing, and loss of natural resources that are central to cultural practices are climate‐driven impacts threatening Indigenous Peoples living in the state. Indigenous knowledges and cultural traditions are important sources of information about natural hazard risks, climate change exposures, and natural resource management practices, especially when collaboratively incorporated into adaptation and resilience responses.

**Key Finding 5: Innovative responses to climate change present opportunities to contribute to equitable and just transitions to sustainability**. Each sector and every community in New York State has the potential to contribute to climate solutions that reduce vulnerabilities, foster resilience, and enhance equity. Local and state governments, schools and universities, nonprofits, museums and cultural institutions, and the private sector all play vital roles in raising climate change awareness, supporting educational and workforce training efforts, and identifying opportunities for innovation that will be necessary to prepare the state for a changing climate. Centering equity in adaptation and resilience actions and aligning these actions with greenhouse gas mitigation strategies is critical for a successful and sustainable climate change response.

BOX 1 **Developments since the 2011 ClimAID assessment**

This chapter substantially expands the scope of social and economic impacts of climate change considered as compared to the 2011 ClimAID study. Whereas ClimAID largely focused on economic impacts in climate‐sensitive sectors such as agriculture, energy, transportation, and natural resources, this chapter broadens the scope to consider impacts on a broader suite of sectors that have not been included in prior assessments, such as local and state government, education, and cultural and historic preservation. This study also pays more attention to vulnerabilities of different population groups, particularly older residents, members of underserved and overburdened communities, Indigenous Peoples, immigrant groups, and LGBTQ+ populations. Finally, this chapter emphasizes opportunities associated with climate change and the need for centering equity as a key element of sustainable climate change response.

Some projections in the 2011 assessment have been borne out by recent evidence. For example, ClimAID projected a shift in freshwater fishing from trout and other coldwater species to bass and other warmwater species. Data from the most recent New York Statewide Angler Survey (2017) demonstrate that the expected shift is already occurring. Similarly, recent studies offer evidence that snow‐ and ice‐related recreational opportunities in New York and the Northeast are being negatively affected by climate change, consistent with ClimAID's projection.

## 2 INTRODUCTION AND BACKGROUND

This chapter explores the impacts of climate change on society and the economy in New York State. It focuses on impacts in five critical areas: (1) populations and migration; (2) the economy; (3) education; (4) culture; and (5) local and state government. The chapter highlights differential vulnerabilities among the state's regions and populations, including climate risks affecting communities, businesses, local governments, and workers. It pays particular attention to issues of equity and environmental justice.

The background material below defines the scope of the chapter and provides context for understanding the social and economic characteristics of New York State. Subsequent sections assess the current literature and data on climate impacts and adaptation as follows:

- Section 3 explores the impacts of climate change in each of the chapter's major topic areas.
- Section 4 considers the vulnerabilities of population groups, communities, regions, and businesses and workers to the impacts discussed in Section 3. Section 4 pays particular attention to equity and environmental justice concerns, examining the vulnerabilities and resilience of environmental justice, Indigenous, and immigrant communities.
- Section 5 identifies opportunities for enhancing adaptation and resilience, emphasizing the need for adaptive actions to explicitly address equity concerns.
- Section 6 looks at opportunities for positive change that can grow out of climate adaptation efforts and identifies emerging topics and research needs in the society and economy sector. This section also provides a conclusion, summarizing the major findings and recommendations presented in the chapter.
- The Traceable Accounts appendix examines each key finding in depth. It provides citations that support each assertion, and it presents the authors’ assessment of confidence in each finding.
- Case studies highlight key climate impacts on New York State's economy and social fabric, along with adaptation and resilience strategies that could serve as models for governments, businesses, and individuals. These case studies are not included in the chapter proper but are available through links provided in the chapter.

The information in this chapter draws from existing literature on the demographic, economic, educational, cultural, and governance impacts of climate change. The authors also collected information through meetings and interviews with dozens of stakeholders representing communities and constituencies from regions throughout the state and through a review of newspaper accounts and policy documents. The assessment of economic impacts of climate change presented here is largely qualitative, drawing attention to key areas and types of impacts as well as critical emerging vulnerabilities. Quantitative estimates of the economic impacts of climate change for each sector will be covered in detail in a separate economic impacts report.

### 2.1 Sector scope and context

The society and economy sector incorporates a broad spectrum of topics, including the demographic, economic, educational, cultural, and governance dimensions of climate change impacts and adaptation in New York State.

#### 2.1.1 Populations and migration

New York State is one of the most socially and demographically diverse states in the country. As of 2020, it had a population of 20.2 million, with 8.8 million living in New York City.^1^ As shown in Table 8‐1, close to 45% of the state's population self‐identifies as Black/African American, American Indian and Alaska Native, Asian, Native Hawaiian and other Pacific Islander, some other race, or two or more races. Roughly 20% of the population identifies their ethnicity as Hispanic or Latino. Nearly one in six New York residents is aged 65 or older, an age group that is one of the fastest growing in the state.^2^ New York State is also home to about 150,000 people who identify solely as Native American and Alaska Native; some belong to the nine federally recognized or state‐recognized Tribal Nations located in New York^3^ (refer to the Assessment Introduction for more information). Native American and Alaska Native residents account for 0.7% of the state's population overall, but as high as 8% of the population in some counties.^1^ As of 2010, nearly 88% of the population of New York State lived in areas classified as urban, with the remaining 12% residing in rural areas.^4^

**TABLE 8‐1:** New York State population in 2020, by race and ethnicity.

Race/ethnicity	Population	Percent
**Race**		
White	11,143,349	55.2
Black or African American	2,986,172	14.8
Asian	1,933,127	9.6
American Indian or Alaska Native	149,690	0.7
Native Hawaiian or other Pacific Islander	10,815	0.1
Some other race	2,210,633	10.9
Two or more races	1,767,463	8.7
**Ethnicity**		
Hispanic or Latino (of any race)	3,948,032	19.5
Hispanic or Latino: white alone	544,442	2.7
Hispanic or Latino: Black or African American alone	227,150	1.1
Not Hispanic or Latino (of any race)	16,253,217	80.5
Not Hispanic or Latino: white alone	10,598,907	52.5
Not Hispanic or Latino: Black or African American alone	2,759,022	13.7

*Note*: Data from U.S. Census Bureau (2020).^9^

With a population drawn from all over the United States and the world, New York is also one of the most diverse states in terms of country or state of origin. The state has historically been one of America's most important gateways for international migrants.^5^ As of 2019, approximately 4.4 million immigrants lived in New York State,^6^ and more than a quarter of the civilian labor force in the state was foreign‐born.^7^ Migration within the state also affects population and settlement patterns. Many domestic migrants have recently left New York City for surrounding counties in the New York City metropolitan region and for other states. Other areas of New York State, such as the Adirondacks, the Champlain Valley, the Great Lakes, and the Central/Finger Lakes, experience seasonal in‐migration during the summer months. The Adirondacks, for example, is home to approximately 130,000 year‐round residents and an additional 200,000 seasonal residents.^8^

In examining the impacts of climate change on populations and migration, this chapter explores regions at risk (Section 3.1.1); impacts on international, interstate, and seasonal migration (3.1.2 and 3.1.3); and population displacement (3.1.4). The chapter also looks in detail at vulnerabilities of particular population groups (4.1), communities (4.2), and locations (4.3).

#### 2.1.2 New York State's economy

New York State's $1.9 trillion economy is the third largest in the country, trailing only the economies of California and Texas.^10^ The largest sector—finance, insurance, real estate, rental, and leasing—accounted for more than 34% of the state's gross domestic product (GDP) in 2021.^11^ Of the 9.4 million employed workers in the state as of 2020,^12^ more than 53% worked for small businesses, which is more than the national average of 46.4%.^13^, ^14^ Health care and social assistance (1.75 million jobs) represented the largest employment sector, followed by local and state government (1.29 million jobs).^15^

As described in the Assessment Introduction, income inequality is prevalent in New York State. The median annual income for all households in the state for the period of 2017−2021 was $75,157.^16^ During the same period, the median annual income was $53,697 for Black or African American households, $55,621 for Hispanic households, and $85,520 for white non‐Hispanic households.^16^ In the third quarter of 2021, the average weekly wage statewide was $1534 in 2021, well above the national average of $1250.^17^ A closer look at the data reveals large disparities in wages between the New York City area and the rest of the state. For example, weekly wages in New York County (Manhattan) averaged $2493 in 2021, while the average weekly wage in many rural counties was less than $1000.^17^

In examining the impacts of climate change on New York State's economy, this chapter focuses on several economic sectors that are directly affected by climate‐related impacts and shocks, including seasonal tourism and outdoor recreation (Section 3.2.1); natural resources, including wood‐related industries and recreational and commercial fishing (3.2.2); and finance, insurance, and investment (3.2.3). Impacts on specific groups, including low‐ to moderate‐income (LMI) households, small businesses, and workers, are discussed in Sections 4 and 5. A full assessment of the economic impacts of climate change in New York State is being conducted as a separate effort.

#### 2.1.3 Education

The education sector has a vital role in every community in New York State. As of the 2020−2021 school year, the state contained 731 public school districts, 4422 public schools, and 351 charter schools,^18^ which altogether enrolled almost 2.5 million K−12 students.^19^ There were also over 1700 private schools in the state,^18^ which enrolled more than 360,000 students in 2020−2021.^20^ In the same period, the state's public and private colleges and universities enrolled more than 1.1 million students in degree‐granting undergraduate, graduate, and professional programs.^21^

The education sector is also a key employer in New York State. There are more than 212,000 public K−12 schoolteachers employed in the state.^22^ In 2020, the state's education sector employed approximately 450,000 people.^15^ Most of the state's K−12 schools are in heavily populated urban and suburban areas. Within these areas, differences in educational quality and educational attainment reflect community‐ and neighborhood‐level wealth inequalities.^23^ In rural areas, many districts have only a single school where all K−12 students are educated. These districts often face acute financial resource pressures due to shrinking populations and local tax bases.^24^

In examining the impacts of climate change on education, this chapter considers impacts on schools and educational delivery (Section 3.3) and on youth (4.1.3). The chapter also discusses the critical role that the education sector plays in informing and educating the state's residents about climate change risks, adaptation, and resilience (5.2 and 6.1).

#### 2.1.4 Culture, arts, and historic preservation

Cultural institutions are vital to both New York State's economy and the identity of New Yorkers. The Museum Association of New York reports that its membership includes more than 750 museums and historic sites ranging from small, local historical societies to the Metropolitan Museum of Art, and from historic forts to one‐room cabins.^25^ In 2020, economic activity in New York State's arts and culture sector accounted for almost $127 billion, corresponding to approximately 7.3% of the state's GDP. In contrast, the sector represents 4.2% of the national GDP.^26^ Museums alone have a total financial impact of $5.37 billion on the state's economy, as they generate 61,796 jobs and $1.38 billion in taxes (2016 values).^27^ In terms of total employment, the arts and culture sector employed more than 435,000 people in 2020,^26^ down from 504,393 in 2019.^28^

This chapter examines how culture, arts, and historic preservation are affected by the impacts of climate change (Section 3.4). It also discusses the vital role that museums and other cultural institutions play in informing and educating the public about climate change (5.2) and bringing about positive change (6.1).

#### 2.1.5 State and local government

The government sector serves critical roles in New York State. These roles include policymaking, public service delivery, public infrastructure investment, land‐use regulation, economic regulation and stimulus, and overall governance at the state and local levels. To carry out effective governance, state agencies work in partnership across executive, legislative, and judicial branches with federal government agencies, counties, towns, cities, villages, school districts, and various special districts like fire protection districts and water and sewer districts. Government agencies also collaborate closely with nongovernmental partners such as nonprofit organizations, private sector and financial institutions, the media, and community‐based organizations. The participation of all these entities and institutions is needed to achieve governance that is effective, efficient, transparent, equitable and inclusive, and responsive and accountable to voters and the general public.

This chapter focuses on the impacts of climate change on state government (Section 3.5.1), local government, and civil society (3.5.2). It discusses the role of government in planning for climate change (3.5.1 and 3.5.2) and in preventing crime, fraud, and price gouging in the wake of climate‐related disaster events (3.5.3). Finally, it looks at the fiscal impacts of climate change on local governments and financial institutions (3.5.4).

### 2.2 Key climate hazards

Given the broad purview of society and economy, nearly all climate hazards affecting New York State will affect some aspect of this sector. Climate hazards of particular concern include worsening flooding, both inland and coastal; rising temperatures and humidity; and changes in the frequency and magnitude of extreme weather events, such as heavy precipitation and heat waves. Other concerns include fluctuating water levels in the Great Lakes, reductions in the length of the snow season, changes in lake‐effect snow events, and changes in the frequency of freeze‐thaw cycles due to increasing temperature variability during winter months. A detailed discussion of climate hazards and climate change in New York State is provided in Chapter 2, New York State's Changing Climate.

This chapter explores how these hazards affect the state's society and economy, with a specific focus on several economic sectors that are directly impacted by climate change (Section 3.2), on specific population groups and communities (3.2 and 4), on state and local governments (3.5), on education (3.3), and on arts and culture (3.4). The economic impacts of climate change on human health and safety, energy, transportation, buildings, agriculture, water resources, and ecosystems are covered in separate chapters.

### 2.3 Nonclimate factors

This chapter acknowledges that climate change is happening within the context of many other large‐scale processes of economic, social, and environmental change, which also act as stressors on New York State's population and industries. For example, economic uncertainties associated with the recovery from the COVID‐19 pandemic and recent geopolitical events, including the war in Ukraine, are contributing to labor shortages, rapid inflation, and fluctuating energy prices. Social challenges confronting the state include rising levels of income inequality; uneven opportunities for social and economic advancement among racial, ethnic, and immigrant groups; and a perception of growing cultural and political divides between rural and urban parts of the state.^29^ Environmental challenges, as outlined in other parts of the report, include development pressures on ecosystems and natural habitats, invasive species, increasing burdens of vectors of disease such as ticks and mosquitoes, and water quality issues, among many others.

The terms “double exposure” and “compound risk” have been used to describe situations where particular regions, communities, groups, or individuals are simultaneously exposed to both climate change hazards and other social, economic, and environmental stressors.^30^, ^31^ Both double exposure and compound risk create added challenges to assessing and managing climate impacts in New York State.

### 2.4 Equity and climate justice

Double exposures within New York State are often a reflection of the state's highly unequal social and economic landscape. High levels of income inequality; differential access to resources across racial, ethnic, and Indigenous groups; and differing assets among rural and urban regions each shape New Yorkers’ vulnerabilities and capacity to respond to climate change impacts. In some instances, as described in the Assessment Introduction, these differential vulnerabilities are also rooted in discrimination associated with policy decisions, disinvestment, and actions such as redlining of urban neighborhoods (a practice where lenders limited or prevented access to credit in areas where large concentrations of Black residents and other racial or ethnic minorities lived).^32^, ^33^ Regional differences are also a legacy of deindustrialization,^34^ which began during the 1970s and was especially devastating to urban neighborhoods in New York City and cities in Western New York, the Mohawk Valley, and elsewhere, leading to large‐scale closure of manufacturing industries and losses of relatively well‐paying manufacturing jobs. Historical land dispossession and forced displacement of Indigenous Peoples have contributed to the vulnerabilities of these groups.^35^

This chapter's exploration of the equity and justice dimensions of climate change in New York State draws attention to differential social, economic, and cultural impacts and vulnerabilities across the state's urban and rural regions, as well as within its demographically and culturally diverse populations. The discussion of vulnerability in Section 4 focuses not only on communities rooted in particular places, such as an urban neighborhood in Buffalo or a rural village in the Southern Tier, but also on vulnerable communities of affinity throughout the state, including immigrant groups, individuals self‐identified as LGBTQ+, and other identity‐based groups.

This chapter directs specific attention to equity and climate justice via individual sections devoted to Tribal Nations and Indigenous Peoples (Section 4.2.1), environmental justice communities (4.2.2), and immigrant communities (4.2.3), as well as through emphasis on equity as a foundation of adaptation and resilience planning, decision‐making, and action (5). The chapter also links to a case study, Adaptation and Equitable Site Remediation, that discusses environmental justice in Buffalo. The Human Health and Safety chapter includes a discussion of how equity and climate justice relate to health care, including reduced access to health care services.

### 2.5 Indigenous communities

Tribal Nations and Indigenous Peoples play a vital role in the economy, culture, and society of New York State. For example, with more than 4000 employees, Oneida Nation Enterprises is one of the largest employers in Madison and Oneida counties and adds more than $1 billion annually to the economies of Madison, Onondaga, and Oneida counties.^36^ This chapter identifies economic and cultural impacts of climate change that are relevant to the Indigenous and Tribal communities in the state, particularly those in the Great Lakes and Atlantic coastal regions, including impacts on natural resources such as timber and fisheries (Section 3.2). It also considers the vulnerabilities of Indigenous Peoples to climate‐related loss of valued species such as ash trees (4.2.1). The chapter recognizes and acknowledges the long history of survival and resilience among Indigenous Peoples and Tribal Nations in New York State. Sections 4.2.1 and 5.1 provide examples of Indigenous Peoples and Tribal Nations’ resilience, including the development of tribal adaptation plans that might offer lessons for other communities in the state.

### 2.6 Opportunities for positive change

Addressing climate change presents many opportunities for state and local governments, the private sector, and civil society to identify innovative responses that contribute to equitable and just transitions to sustainability.^37^, ^38^, ^39^ It also creates opportunities to advance sustainable development, economic resilience, and equity.^38^ For example, efforts to support ecological restoration or urban green infrastructure to address excessive precipitation and flooding issues can also create entrepreneurial opportunities for community members to develop new small businesses and new career tracks for the underemployed. All economic sectors and communities in New York State have the potential to contribute to climate solutions that reduce vulnerabilities and enhance adaptation. Section 6.1 provides an overview of emerging opportunities to promote sustainable and just transitions identified during the assessment process in areas such as supply chains, education, and workforce training. The chapter also links to the Adirondacks as a Cycling Destination case study, which describes how the Adirondacks region is using the development of new bicycling opportunities as an emerging form of economic adaptation to climate change.

## 3 IMPACTS

This section describes the impacts of climate hazards on each of the major areas of the sector: populations and migration, the economy, education, culture and the arts, and local and state governments and civil society. Rather than focusing on specific climate scenarios, the section considers the general impacts of current and future climate hazards. These hazards include worsening flooding, rising temperatures and humidity, changes in the frequency and magnitude of extreme weather events such as heavy precipitation and heat waves, and reductions in the length of the snow season.^40^ The section considers impacts for New York State as a whole, as well as impacts for specific regions and communities, while also recognizing the differences that exist across the state in terms of income, employment, and capacity to recover from shocks and stressors. Returning to the notion of double exposure, many of the impacts discussed here result from intersections between climate change exposures and other types of social and economic stressors affecting New Yorkers. Examples of these stressors include rising levels of income inequality, racial discrimination, rising energy costs, inflation, and supply chain disruptions. Each of these stressors can interact with and exacerbate climate change impacts, with particularly severe consequences for already vulnerable populations, communities, businesses, and workers, as will be discussed in Section 4.

### 3.1 Impacts on populations and migration

The impacts of climate change in New York State are highly uneven across different groups, communities, and regions. This section describes regions of the state at risk from different types of climate hazards, the potential impacts on international and domestic migration, and concerns about population displacement. A more in‐depth look at the vulnerabilities of different population groups, communities, and regions is provided in Section 4. Opportunities for enhancing the resilience of vulnerable groups and promoting just transitions are discussed in Section 5.

#### 3.1.1 Regions at risk

Regional exposures to climate hazards vary by hazard type and population characteristics. This section focuses on several specific climate hazards that are affecting New York State: hurricanes, coastal flooding, inland flooding, and winter weather. Patterns of heat exposure are described in the Human Health and Safety chapter. The discussion in this section focuses on the Federal Emergency Management Agency's (FEMA's) National Risk Index.^41^ This risk index calculates risk based on the historical frequency of several climate hazards, but does not consider how climate change may alter the risk of these hazards from their historical frequency.

As Figure 8‐1 shows, hurricane frequency is greatest on Long Island and in New York City. These areas are expected to experience a hurricane once every 7 years, on average, according to estimates provided in FEMA's National Risk Index. The neighboring Hudson Valley also has comparably high exposure to hurricanes. These areas with the highest level of hurricane risk are the most populous parts of the state and are projected to remain the most populous parts going forward to 2050, regardless of what future population scenario is realized. Hurricanes can also impact other regions of the state, even if the frequency of events in those areas is expected to be lower. In 2011, for example, Hurricanes Irene and Lee caused severe damage across many parts of New York. There is increasing confidence that the intensity and rainfall of tropical cyclones like hurricanes will increase in the future.^40^

**FIGURE 8‐1:**
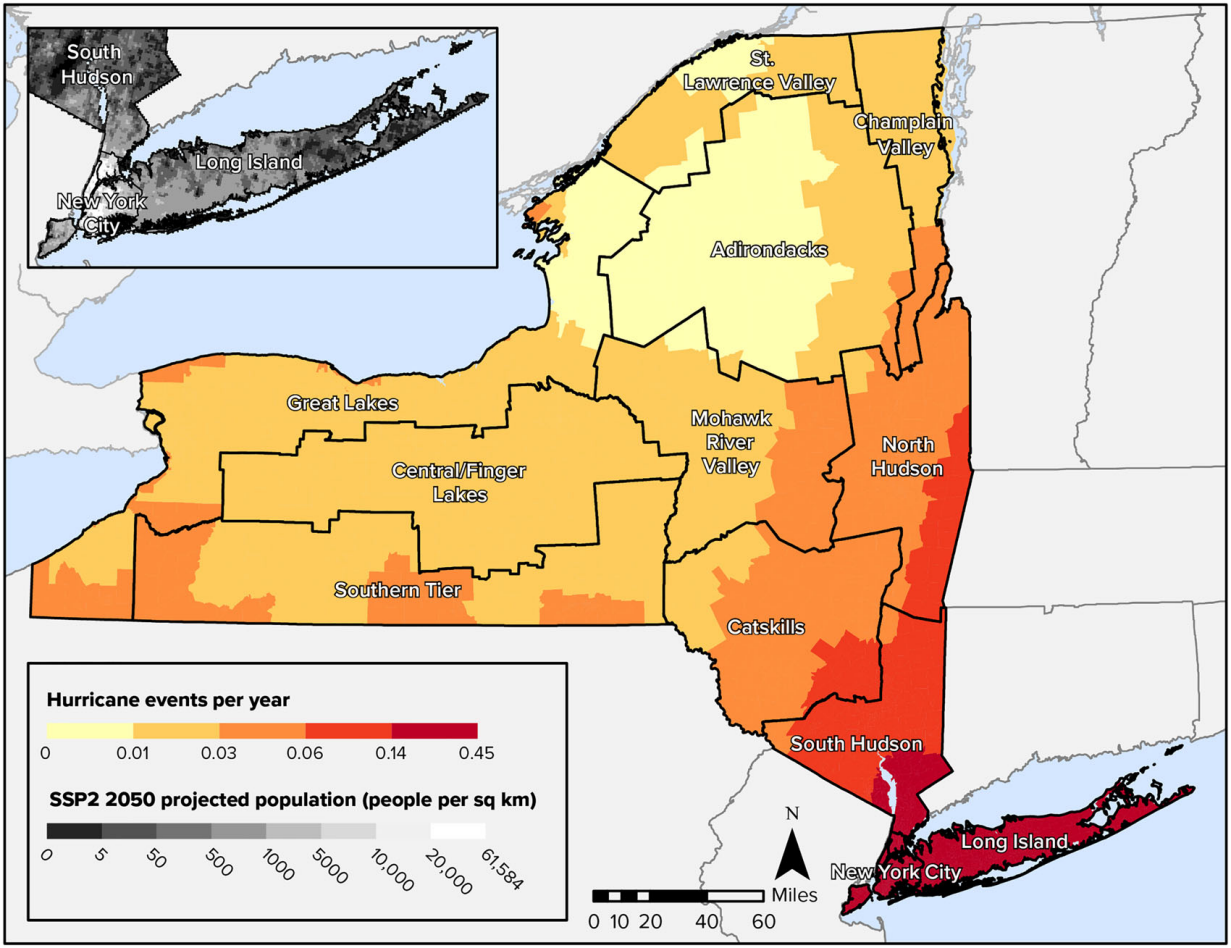
Average number of hurricane events per year in New York State, 1851–2020, with projected 2050 population density shown for the highest‐risk areas. Data from FEMA (2023)^42^ and Zoraghein and O'Neill.^43^

##### 3.1.1.1 Coastal flooding

In 2021, nearly 20 million people lived in New York State counties adjacent to the ocean or Great Lakes. In 2021, these counties employed 8.8 million people who earned wages totaling more than $776 billion. These areas generated $1.9 trillion in GDP in 2021.^44^ New York's exposure to sea level rise and ocean‐related coastal hazards is among the highest of all U.S. states. New York contains five of the 20 U.S. counties with the highest population in the low‐elevation coastal zone (Kings, Nassau, New York, Queens, and Suffolk counties)—that is, in areas that are contiguous to the seacoast and less than 32.8 feet (10 meters) above sea level.^45^

As shown in Figure 8‐2, exposure to coastal flooding in New York is concentrated in New York City, the surrounding counties in Long Island, and the lower Hudson Valley. More than 3 million New Yorkers live within the low‐elevation coastal zone.^45^ A study of six community districts in New York City's five boroughs found that recent updates of the FEMA 100‐year floodplain boundaries, along with changes in land use, have increased the extent of the floodplain in these districts by nearly 46% since 2007.^46^ This increase has exposed 10.5% more New York residents—and 7.5% more people from vulnerable population groups—to the risk of coastal flooding. Further discussion of the vulnerabilities of Atlantic coastal communities can be found in Section 4.3.3.

**FIGURE 8‐2:**
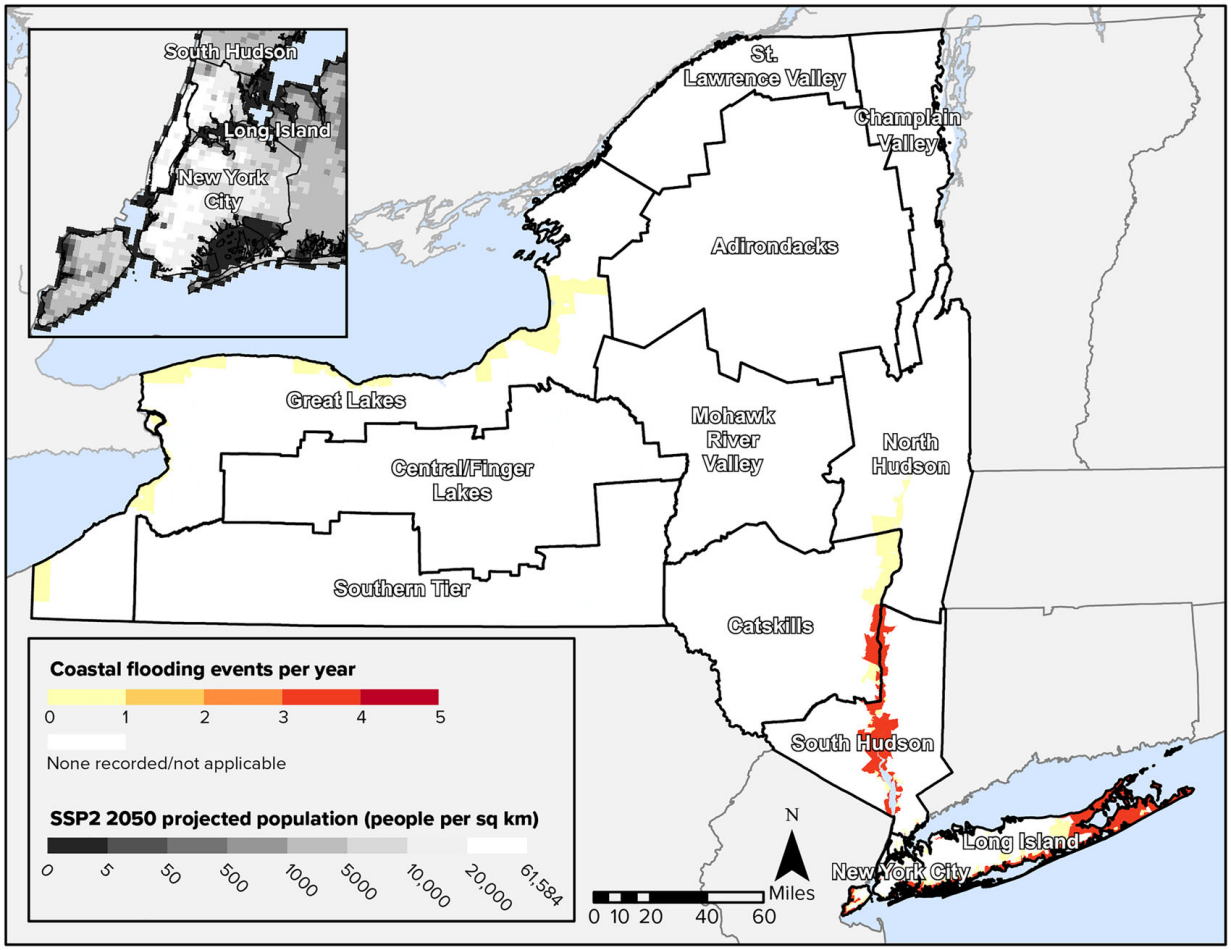
Average number of coastal flood events per year in New York State, historically, with projected 2050 population density shown for the most populous at‐risk area. Date ranges for the historical data used in calculating averages differ based on location and type of flood hazard present.^47^ Data from FEMA (2023)^42^ and Zoraghein and O'Neill.^43^

##### 3.1.1.2 Inland flooding

Exposure to riverine or inland flooding, shown in Figure 8‐3, is widely distributed throughout the state. The southern portion of the Hudson Valley has the most severe exposure, experiencing more than four flood events per year on average. Long Island experiences three to four flood events annually, as do some localities in nearly every region. This wide distribution includes census tracts in urban, suburban, and rural localities—from Buffalo to Binghamton. The wide‐ranging distribution of inland flooding has implications for how the state should prepare for and adapt to future flood conditions. The projected increase in heavy rain events makes inland flooding events more likely in the future.^40^ Depending on how the state's population distribution changes in the future based on migration and other factors, the number of people exposed to inland flooding could grow in some regions.

**FIGURE 8‐3:**
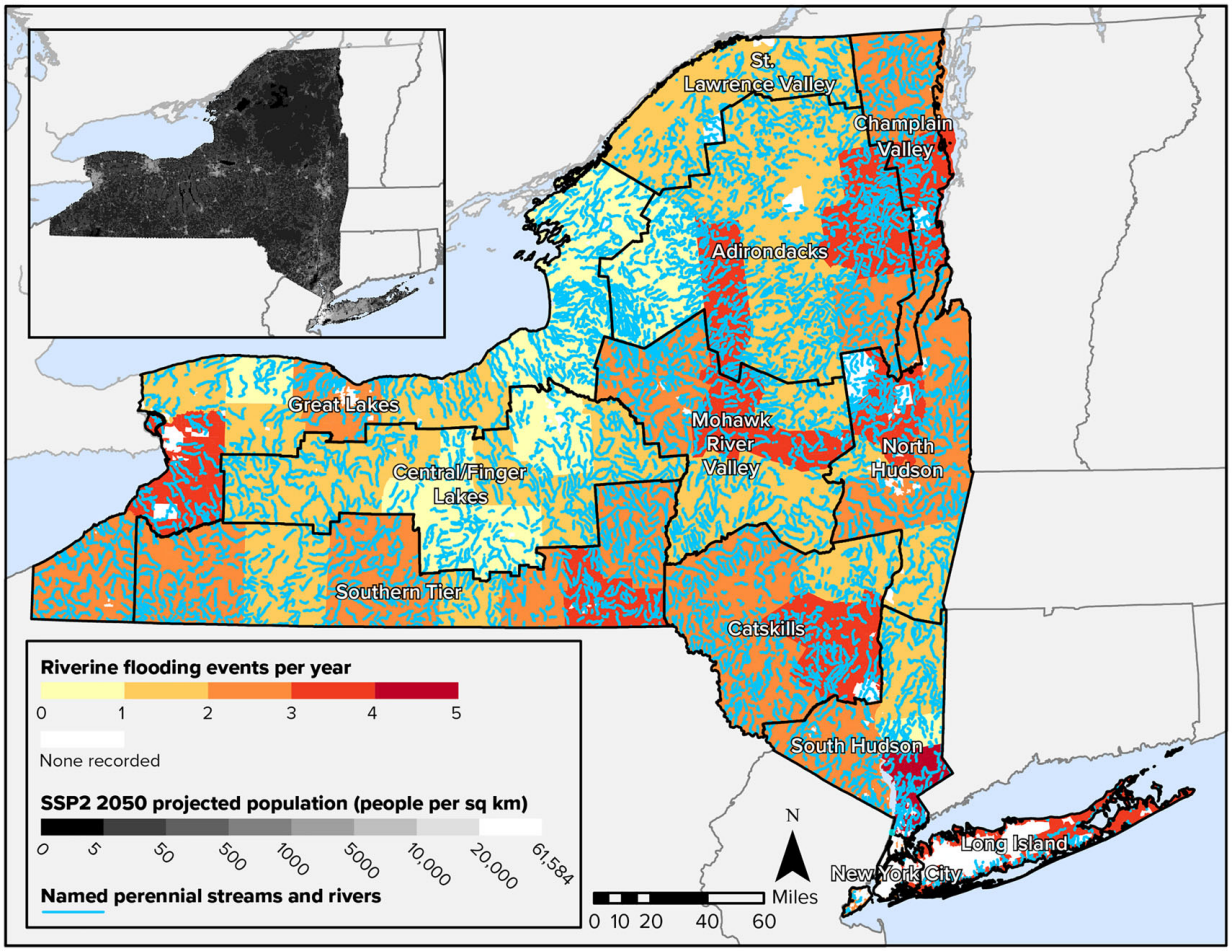
Average number of riverine flooding events per year in New York State, 1996–2019, with projected population density for 2050. Data from FEMA (2023)^42^ and Zoraghein and O'Neill.^43^

##### 3.1.1.3 Severe winter weather

Figure 8‐4 shows the spatial distribution of winter weather events in New York State. FEMA defines a winter weather event as a winter storm event “in which the main types of precipitation are snow, sleet, or freezing rain.”^41^ The Great Lakes region has the most severe winter weather, with many localities experiencing more than 18 events per year. Some parts of the Adirondacks experience a similar number of events, as do communities in the Southern Tier near Lake Erie. While the impact of winter weather ripples through daily life, disrupting school hours and commutes, future winter weather may take its greatest toll on older adults. The population of people 65 or older is one of the fastest‐growing age groups in the state, and there are relatively high concentrations of older adults in many areas that have the most frequent winter weather events, including in some counties of the Adirondacks and in Chautauqua County, southeast of Lake Erie. Other regions of the state that have historically experienced 9−12 winter weather events per year (parts of the Central/Finger Lakes region, for example) also have high proportions of older adults. When major winter storms hit these areas, there are likely to be increasing demands on the provision of services such as reliable heat and electricity—particularly for older adults, many of whom are isolated.

**FIGURE 8‐4:**
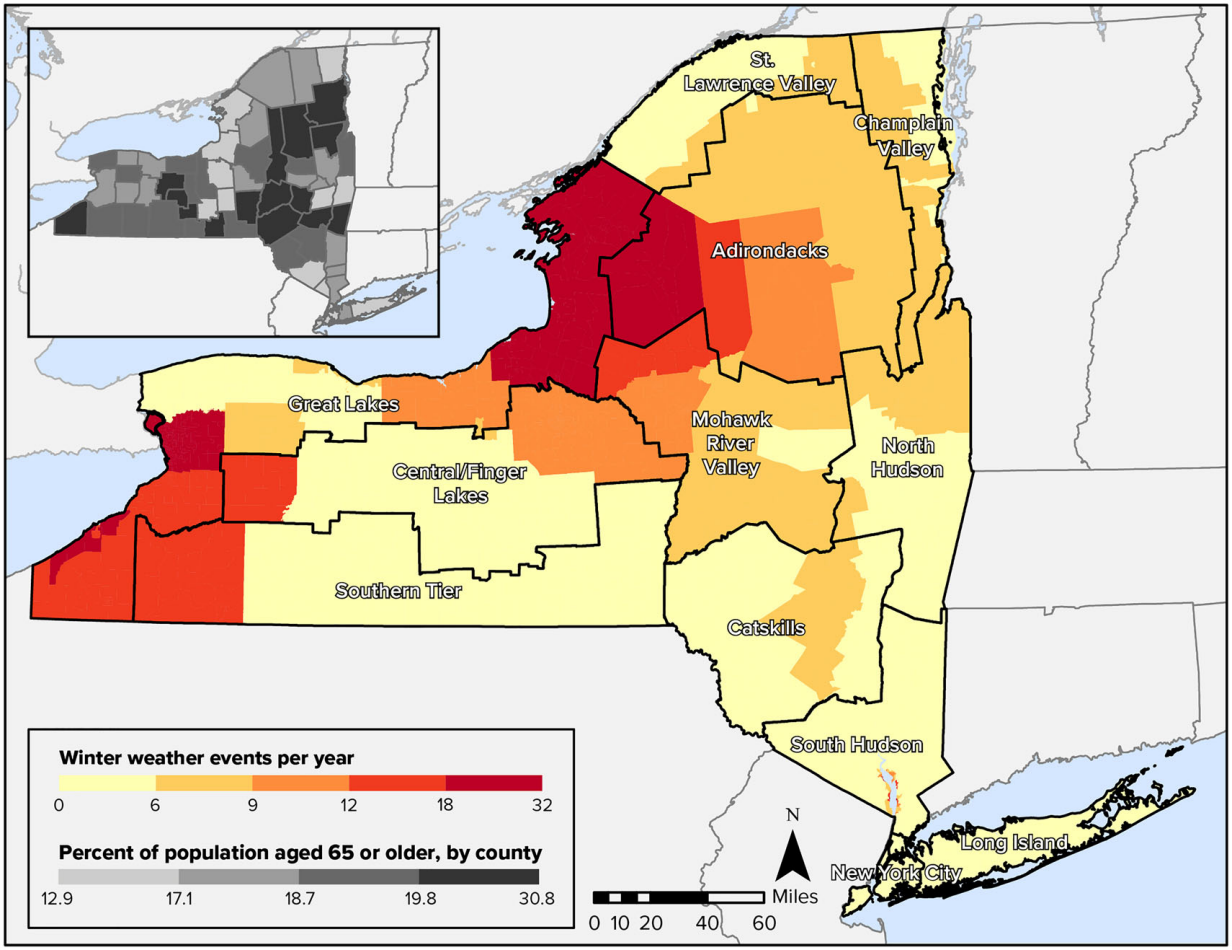
Average number of winter weather events per year in New York State, 2005–2021, with estimated percentage of population aged 65 or older, 2016–2020. In the scale for the inset map, each subgroup (e.g., 12.9−17.1%) contains approximately one quarter of the counties in the state. Data from FEMA (2023)^42^ and U.S. Census Bureau (2020).^48^

#### 3.1.2 International and interstate migration

New York State has traditionally been a major destination for immigrants to the United States. Today, 4.4 million immigrants live in the state, and more than a quarter of the labor force is foreign‐born. According to the American Immigration Council, as of 2018, the most common countries of origin for immigrants to New York State were the Dominican Republic (11% of immigrants), China (9%), Mexico (5%), Jamaica (5%), and India (4%).^5^ Countries of origin in New York City are unusually diverse for the United States, with no one group, region, or even language constituting a majority.^49^

Climate change is expected to have substantial impacts on migration patterns globally. However, separating the climate‐related causes from economic, political, and other causes of migration is difficult. To date, evidence of international migration as a direct result of climate disruption is limited.^50^ Within the United States, domestic migration data show that populations continue to move toward areas that are highly exposed to climate hazards, including coastal cities and regions that are vulnerable to hurricanes and coastal storms, arid regions that are vulnerable to water stress, and wildfire‐prone regions.^51^ All these regions are expected to experience increasing exposure to climate hazards in the future, potentially putting many of these new populations at risk.

Several studies have attempted to model the potential migration patterns of people displaced by future sea level rise. Modeling studies have suggested that with more precise sea level scenarios, it may become easier to predict the domestic migration that will result from people leaving areas projected to experience sea level rise.^52^ Among the plausible futures is one where the population of New York State declines by midcentury and continues to decline because of low international *and* domestic migration.^53^

To date, there is little evidence of widespread migration into New York State from areas with sea level rise exposure. Nevertheless, it is possible that the state could be a destination for climate‐displaced groups and individuals in the future, particularly from countries and regions where existing migration networks to New York are already in place. Recent work by the American Society of Adaptation Professionals has considered the possibility that the Great Lakes region could become a climate refuge, partly due to its cooler temperatures and access to fresh water.^54^, ^55^ Within New York State, Buffalo received a few hundred migrants who were displaced by Hurricane Maria in 2017,^56^ and the city is taking action to prepare itself to receive more climate migrants.^57^ However, evidence of climate‐related migration in New York State remains largely anecdotal,^58^ and some question the validity of the idea that the Great Lakes region could become a climate haven, pointing out that the region already faces numerous issues that could be exacerbated by climate change, including problems with flooding, agricultural pollution, and invasive species.^59^

#### 3.1.3 Seasonal migration

Areas such as the Adirondacks, Lake Champlain, the Finger Lakes, and Thousand Islands experience seasonal migration, characterized by an influx of residents during the summer months and an outflow of residents during the winter months. Summer residents make vital contributions to the local economies and tax bases of those areas. In some places, the seasonal summer population is nearly twice the size of the year‐round population. The Adirondacks, for example, is home to approximately 130,000 year‐round residents and an additional 200,000 seasonal residents.^8^

Climate hazards such as flooding, rising temperatures and humidity, and changes in precipitation and the magnitude of heat waves may affect seasonal migration patterns. There is a possibility that climate change could bring more seasonal residents to areas such as the Catskills, Finger Lakes, and Adirondacks. While such influxes might be beneficial from a net economic standpoint, they can also add to the existing housing challenges in many regions, as discussed in Section 4. The Adirondacks region, for example, is already facing a regional housing crisis due to unregulated seasonal rentals that are displacing younger residents.^60^

Short‐term projections (i.e., through 2040) suggest that in many New York counties with growing populations of older adults, the number of people moving into the area will exceed the number moving out.^61^ Changes in temperature patterns could affect the seasonal migration of retired people who spend the winter months in locations such as Florida and Arizona (both of which are becoming hotter during both spring and fall). While this issue requires additional study, warmer spring and fall temperatures in New York State could lead these “snowbird” migrants to arrive earlier in the spring to their summer homes in New York and remain longer into the fall season before returning south. Patterns of seasonal migration to the state's rural agricultural communities could also change due to changes in seasonal labor demands, driven by longer or altered growing seasons or changes in the types of crops produced.^62^

#### 3.1.4 Population displacement

Within New York State, climate change may also contribute to population displacement. Displacement is a form of migration in which individuals are forced to move against their will. In New York City, rising rents and gentrification pressures are contributing to displacement and local out‐migration of low‐income residents.^63^ Within the metropolitan area, these populations are moving to outer boroughs, such as Queens, or to first‐ and second‐ring suburbs. Direct and indirect climate change impacts may also potentially contribute to population displacement within the state, particularly from flood‐prone and low‐lying coastal areas, some of which are already experiencing “sunny day” flooding as discussed in Section 4.3.3. In some areas, concerns about climate‐related displacement are further magnified by the existing gentrification pressures that low‐income communities already face (refer to Section 4.2.2). As discussed in Section 4.2.2, displacement concerns can also exacerbate the vulnerability of low‐income households to extreme climate events and related hazards. Detailed discussion of population displacement and adaptive responses such as buyout programs can be found in the Human Health and Safety chapter.

### 3.2 Economic impacts

Climate change is expected to have serious economic impacts in New York State.^50^, ^64^, ^65^ Nearly every economic sector in the state is directly or indirectly affected by climate change. Economic sectors that are climate and weather‐dependent, such as outdoor recreation and tourism, may experience substantial direct impacts as temperature and precipitation patterns change and extreme weather events such as flooding and heat waves become more frequent. For sectors such as manufacturing, wholesale and retail, insurance, and finance, the impacts of climate change may be more indirect, hitting the bottom line via disruption of energy, transportation, and supply chains. These sectors may also experience increasing climate‐associated risks, higher claims for weather‐related damages, and climate‐related damage to investment properties and businesses.

Interdependence between sectors and within a globalized economy presents both considerable risks and potential opportunities for businesses in New York State. For example, manufacturers of complex products that rely on global labor and supply chains of imported parts may halt production when climate events disrupt operations and shipping in a geographic region. As illustrated by supply chain disruptions associated with the COVID‐19 pandemic, the state's retail, wholesale, and manufacturing sectors are vulnerable to production shutdowns in overseas plants and disruptions to international shipping.

Cross‐state and international connections are crucial to New York State's economy. The economy and the labor market of New York City, for example, are part of a highly integrated metropolitan regional economy that includes portions of New Jersey and Connecticut. Buffalo and Niagara Falls have economic connections to the cities across the border in Ontario. Beyond these spatial linkages, New York State's economy also relies heavily on international and domestic trade, supply chains, and labor.

Climate change impacts, whether direct or indirect, could damage underlying economic structures and result in substantial economic and personal harms or risks. The COVID‐19 pandemic had a profound impact on New York State's employment, as illustrated by the loss of more than 955,000 jobs during 2020.^66^ Similar to the COVID‐19 pandemic, climate change could disproportionately impact specific economic sectors in the state, including the leisure and hospitality sector, which lost more than one‐third of its employment during 2020.^67^ Other sectors that may experience substantial job losses include the construction sector and the trade, transportation, and utilities sector, both of which lost more than 10% of their jobs during the pandemic. In contrast, the manufacturing, education, and professional and business services sectors each lost more than 5% of their jobs.^67^ These sectoral impacts can occur through numerous channels from direct storm‐related damage to plants and facilities, to power outages that disrupt operations, to widespread shifts in consumer preferences. This section considers the impacts of climate change on several climate‐sensitive economic sectors, including outdoor tourism and recreation, natural resources, and finance and insurance. Economic impacts in other climate‐sensitive sectors, such as agriculture, are covered in other chapters of this assessment. Opportunities for enhancing economic resilience in New York State are discussed in Sections 5.3 and 6.1.

#### 3.2.1 Outdoor tourism and recreation

Tourism (both outdoor tourism and other tourism) plays a significant role in the economy of nearly every region of the state. From the Statue of Liberty in New York City to Niagara Falls, New York's tourist attractions brought more than 250 million visitors to the state in 2019.^68^, ^69^, ^70^ State tourism levels plummeted in 2020 due to the COVID‐19 pandemic. New York City was hit particularly hard, with the number of visitors declining by approximately two‐thirds compared with 2019. Tourism activity began to recover during 2021 and, as of this writing, is expected to show recovery during subsequent years.^71^ Tourism spending in New York State totaled more than $73 billion in 2019.^68^ Figure 8‐5 shows how this spending breaks out by tourism region. An estimated 10% of this spending (i.e., $7.3 billion) was on recreational activities, of which outdoor‐focused tourism and recreation are important components. While employment in New York City's tourism sector is year‐round and often unionized, the tourism sector in many other parts of the state is often seasonal and low‐wage, particularly in many of the state's rural and coastal communities. Within the Adirondacks and the Catskills, tourism accounts for 19% and 17% of employment, respectively—the highest proportions in the state.^68^

**FIGURE 8‐5:**
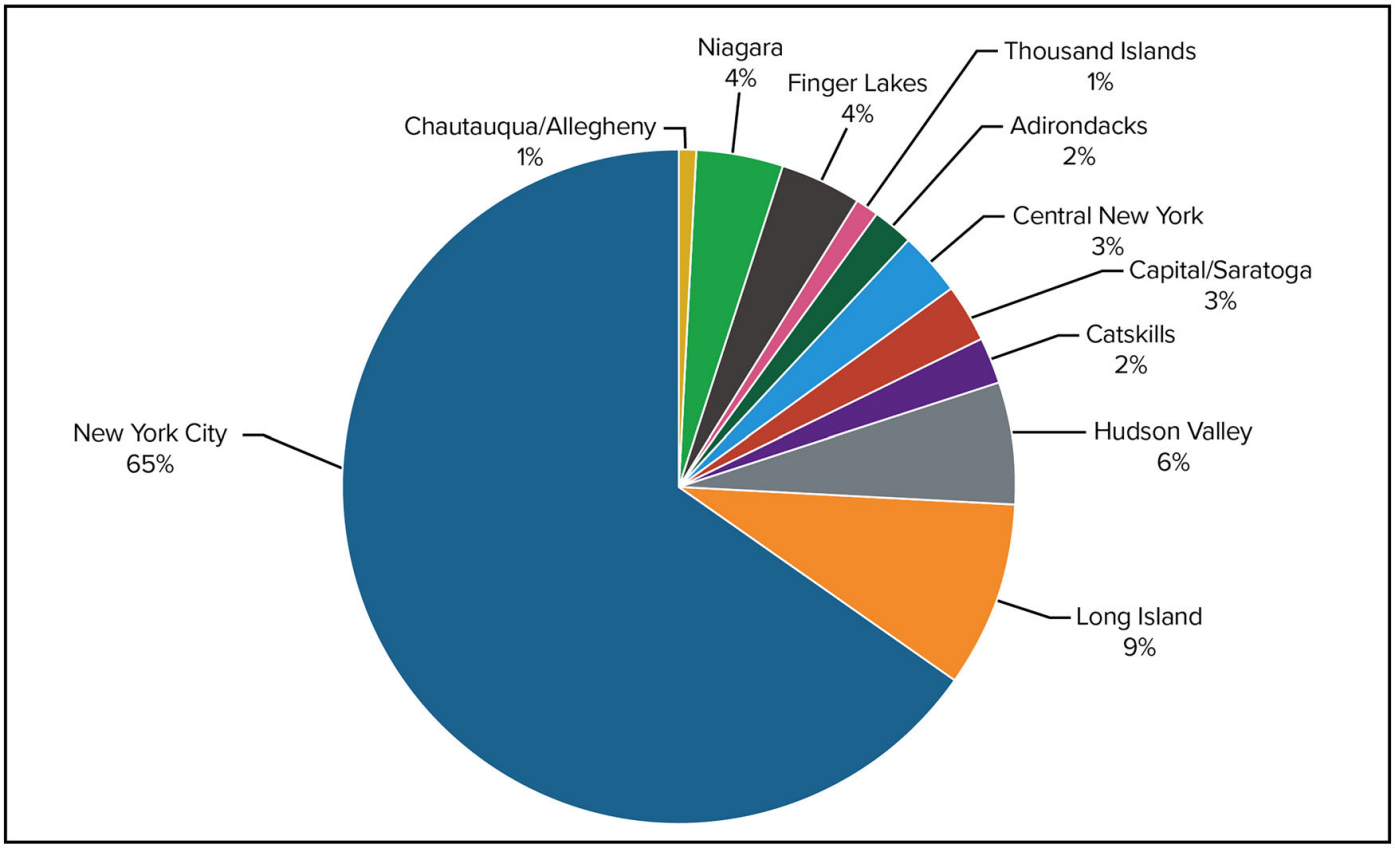
Share of New York State tourism spending by region, 2019. Data are broken out by regions as defined within the Tourism Economics report. Data from Tourism Economics (2019).^68^

Compared with other states, New York's outdoor recreation sector—a component of the broader tourism sector discussed above—is among the largest in terms of employment and value‐added. In 2021, the sector employed more than 248,000 people (241,000 in 2020 and 291,000 in 2019) and produced more than $25 billion in GDP (over $21 billion in 2020 and $29.2 billion in 2019).^72^, ^73^, ^74^ Figure 8‐6 presents the breakdown of GDP in New York by major outdoor recreation activity.

**FIGURE 8‐6:**
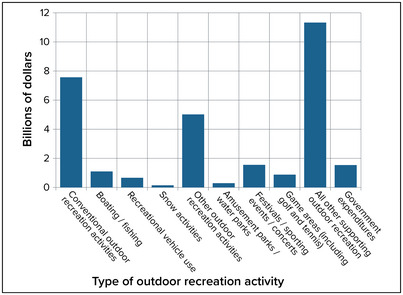
GDP by New York State outdoor recreation activity, 2021. Category totals add up to more than the state total of $25.5 billion because of some overlap across categories. Data from U.S. Bureau of Economic Analysis (2022).^74^

The outdoor tourism and recreation sector in New York State is not only dependent on public health and economic conditions but also directly linked to seasonal expectations about weather and climate. Because of this, it is highly sensitive to climate change impacts. In particular, climate change may affect the timing and availability of seasonal recreational activities such as skiing, boating, and leaf‐peeping. All these activities play critical roles in the local economies of outdoor tourism−oriented areas of the state, including many communities in the Great Lakes, Central/Finger Lakes, Adirondacks, Catskills, Mohawk River Valley, North and South Hudson, Champlain Valley, and Long Island assessment regions.

##### 3.2.1.1 Winter tourism

Snow‐related recreation and tourism are important sources of revenue within the Adirondacks, Catskills, Central/Finger Lakes, and Great Lakes assessment regions. The negative impacts of climate change on traditional winter recreational sports in New York State are expected to be profound. As highlighted in the ClimAID report, changes in the timing and amount of snowfall and the length of the winter season affect snow‐related recreation including skiing, snowmobiling, and snowshoeing.^64^, ^75^

Recent studies offer evidence that snow‐related recreational opportunities in New York and the Northeast are being negatively affected by climate change.^76^, ^77^, ^78^, ^79^ One study found that 125 ski resorts in New York State closed between 1969 and 2019, and identified changing climate conditions as playing a role in many of these closures.^80^ Another study documents how climate change has reduced the ability of ski areas in the Northeast to maintain a 100‐day ski season and to open for the economically important Christmas‐New Year holiday period.^76^ A third study models changes in the number of days available for downhill skiing and snowmobiling in several states under two climate change scenarios (SSP2‐4.5 [intermediate greenhouse gas emissions] and SSP5‐8.5 [very high greenhouse gas emissions]).^77^ As shown in Table 8‐2, the study projects significant declines from the historical averages of 68 days for downhill skiing and 101 days for snowmobiling in New York State.

**TABLE 8‐2:** Days per season suitable for downhill skiing and snowmobiling under two climate change scenarios.

Scenario	Time period^a^	Days suitable for downhill skiing	Days suitable for snowmobiling
N/A	Historical	68	101
SSP2‐4.5	Near future (2020s)	50	82
	Mid future (2050s)	42	72
	Far future (2080s)	36	65
SSP5‐8.5	Near future (2020s)	49	81
	Mid future (2050s)	31	59
	Far future (2080s)	18	39

*Note*: Data from Chin et al.^77^

Abbreviation: SSP, shared socioeconomic pathway. Refer to New York State's Changing Climate for further discussion on climate scenarios.

^a^ According to Chin et al., “The near future period is based on climate projections for a 30‐year window centered on the 2020s (2011−2040); the mid future period on climate projections for the 2050s (2041−2070); and the far future period on climate projections for the 2080s (2071−2100).”

Ski resorts are already using advanced snowmaking technology in response to unreliable natural snowfall. However, according to research from the University of New Hampshire, given the low temperatures needed to produce snow, at some point the technology will not be able to keep up with rising temperatures, and many more ski resorts may be forced to close. As in New York State, many ski resorts in New England have already closed.^80^ By the end of the century, the study anticipates that only 15% of resorts in New England and southern Quebec—which face similar climate conditions and challenges to New York—will be able to remain operational.^81^

As climate change adversely affects winter industries, including ski tourism, it might also present opportunities to promote diversity and inclusion in sports frequently lacking participation from communities of color, as well as low‐income populations and other vulnerable groups. This can be achieved by partnering with community‐based and nonprofit organizations focused on diversity, inclusion, and racial justice. For example, the resort company Powdr, in response to the increase in costs associated with artificial snow production and shortened snow seasons, introduced the “Play Forever” campaigns in partnership with Stoked, a nonprofit that mentors young people from underserved communities who are interested in board sports.^82^

##### 3.2.1.2 Summer tourism

The impacts of climate change on summer tourism in New York State are likely to be mixed. On the positive side, warmer summers and longer spring and fall shoulder seasons may increase participation in and the numbers of days available for many types of warm‐weather outdoor recreational activities. One study projected that warmer summers along the Atlantic Coast could result in increased participation in waterfront‐related recreational activities, but the study cautioned that the magnitude of the projected positive impacts is highly dependent on assumptions about how weather affects behavior.^83^ Warmer fall and spring temperatures could also extend the summer boating season in the Great Lakes, the Adirondacks, Long Island, and other regions.

Beach erosion due to sea level rise along the Atlantic Coast could offset the positive impacts of warmer temperatures on waterfront activities. A study of the impacts of changing beach widths due to erosion in the nearby Delaware Bay region found significant reductions in beach visitation associated with the narrowing of beaches.^84^ Studies have also documented the possibility that sea level rise could cause significant erosion or damage at beaches in New York City and other areas of coastal New York.^85^ Such changes may reduce interest in and opportunities for summer beach recreation and tourism. In the Great Lakes, fluctuations in lake levels may affect marinas and boating opportunities, with economic consequences for summer tourism−focused communities (refer to Section 4.3.2 and the Village of Sodus Point Water Level Adaptations case study for more information).

Another concern for water‐focused recreation is the increasing frequency of harmful algal blooms (HABs) in lakes and tidal areas, as illustrated by the effect of the intense 2018 “red tide” events in Florida.^86^ In New York State, HABs have led the Department of Environmental Conservation to recommend caution when swimming, drinking water, or fishing in parts of Lake Ontario, the Finger Lakes, and Lake Champlain, among other locations.^87^ The Department has also warned that pets, livestock, and other animals can be at risk if exposed to HABs. HABs that occurred in Lake Ontario in 2010 caused vacationers in Sodus Point to cancel lodging reservations and leave the area, resulting in economic hardships for local businesses.^88^ The growing presence of invasive aquatic species is also a concern as it may affect revenues associated with boating and related water activities in the Hudson River, Adirondacks, Central/Finger Lakes, Great Lakes, and elsewhere.

Refer to the Ecosystems and Water Resources chapters for more information on HABs and their relationship to climate change.

##### 3.2.1.3 Fall tourism

Fall season recreational tourism is also likely to be affected by climate change. Changes in the timing or duration of the fall foliage season^89^ could affect leaf‐peeping tourism in the Hudson Valley, Catskills, Adirondacks, Finger Lakes, and other areas. Some studies suggest that warmer temperatures may delay the onset of fall color changes, leading to a shortened foliage season, and an increase in the number of strong storms could cause earlier leaf drop. Although a longer period of monitoring is needed to confirm any trends, recent observations align with these projections and show increasing year‐over‐year variability in the foliage season.^89^, ^90^ Seasonal changes are also affecting mammal and bird behavior and habitat, with implications for subsistence and recreational hunting species such as deer, bear, geese, and turkeys.^91^ Changes in species behavior will also have direct impacts on the Indigenous Peoples living in the state who depend on various species for subsistence livelihoods and/or cultural traditions. Changes in the timing of the leaf‐peeping and hunting seasons will require adaptations by communities that depend on tourism associated with these activities.

Another fall (and summer) recreational activity that is likely to be affected by a changing climate is bicycling. Cycling is growing in visibility and popularity in many areas of New York State. Within the Hudson Valley and the Catskills, for example, cities such as Poughkeepsie and New Paltz sit at the junction of numerous cycling trails that are attracting growing numbers of cycling tourists. Warming summer temperatures along with warmer spring and fall seasons could increase interest in and opportunities for cycling in the state. Within New York City, a study of the impacts of climate change on bike share usage found that usage increases with rising temperatures and projected increases in cycling under both the SSP2‐4.5 and SSP5‐8.5 scenarios for the period from 2040 to 2069.^92^ The study noted that decreased cycling in summer due to increased frequency of very hot days would be offset by increases in cycling during other seasons.^92^ Another study, which focused on the effects of climate change on weekend usage of bike‐share programs in 16 North American cities, including New York City, found that a warming climate will have significant positive effects on cycling demand.^93^ The Adirondacks as a Cycling Destination case study describes efforts to expand cycling opportunities in the Adirondacks and provides further discussion of cycling expansion as an adaptation to climate change.

While adaptation to the impacts of climate change will be critical for the viability of outdoor tourism in New York State, it is also important to recognize that changes in tourism volume can have unintended negative consequences on sensitive ecological areas and ecosystems. For example, local Adirondack organizations have expressed concern about the impacts of increased outdoor recreation on the climate resilience of the region's ecosystems, particularly during ecologically sensitive spring and fall “shoulder” seasons that historically have had fewer visitors. These ecosystems already face stress from increased human disturbance.

#### 3.2.2 Natural resource sectors

Natural resource−based sectors, including forestry and fisheries, and the ecosystem services they provide, are important for the state's rural and coastal regions and communities, including its Indigenous Peoples. Natural resource sectors and ecosystem services are also highly sensitive to climate change. The impacts of climate change on the state's ecosystems are discussed in detail in the Ecosystems chapter.

##### 3.2.2.1 Wood‐related industries

The $13.1 billion wood‐related industry in New York State includes four main categories of economic activity: forestry and logging ($302 million), wood products ($2 billion), pulp and paper ($9.3 billion), and wood furniture ($1.5 billion).^94^ In 2014, the industry generated more than $2.5 billion in payroll for nearly 41,000 jobs, evenly distributed across the state.^94^ Long Island and New York City have more than 10,000 direct jobs in wood products, pulp and paper, and wood furniture making. Forestry is also an important contributor to the economies of the Southern Tier, the southern portion of Western New York, and the North Country region (which are defined as state economic development council regions and are slightly different from the assessment regions).^94^ The forest economy is of particular importance in the North Country (i.e., parts of the Adirondacks, St. Lawrence Valley, and Champlain Valley assessment regions), where jobs directly and indirectly connected to this sector represent more than 4% of all industry jobs in the region.^94^

Forest ecosystem services include providing food, fuel, and fiber; cleaning the air; filtering water supplies; regulating floods and erosion; supporting biodiversity; and reducing the climate impacts of greenhouse gas emissions by sequestering and storing carbon. About 55% of New York State's land area is classified as forest^91^; 74% of New York State's forests are privately owned.^95^

Climate change exacerbates several factors affecting forest quality and forest regeneration.^96^ Warmer winter temperatures and decreased snow cover are favorable to invasive species and other insect pests that are already devastating hemlock, ash, and other tree species. Currently, deer and invasive plant species are impeding the normal regeneration of forests—a situation likely to worsen as these species are expected to thrive in a changing climate.^97^

Earlier snowmelt in the spring and longer growing seasons combined with warmer temperatures may lead to more frequent moisture stress during the growing season, which can be particularly harmful to younger trees. Changing precipitation patterns may exacerbate this stress, especially if summers feature heavy rain events with relatively dry periods in between. Forest fragmentation may hinder the movement of plant and animal species through forests while increasing susceptibility to invasive species.^95^ The specific impact climate change may have on the timber industry is uncertain.

Of the tree species that may be impacted by climate change (e.g., black ash, balsam fir, paper birch, northern white cedar, sugar maple),^97^ many have important economic and profound cultural and spiritual significance for Indigenous Peoples.^98^, ^99^ Black ash, for example, is threatened across New York State by the invasive emerald ash borer, the range of which will likely spread with warming temperatures.^100^ The Haudenosaunee have a rich tradition of making fancy and utilitarian baskets from ash splints. Disappearance of the ash trees would disrupt both culture and business.^101^

In addition to having substantial economic impacts, changes in forest composition will also have profound nonmaterial impacts for Indigenous Peoples, including the potential loss of culturally meaningful winter and summer traditions and activities. Sections 4 and 5 provide additional discussion of vulnerabilities and identify creative adaptation strategies.

##### 3.2.2.2 Recreational fishing

Rising temperatures and reductions in habitat for coldwater species^91^ are expected to have substantial impacts on New York State's recreational fisheries. The state's freshwater fisheries generate nearly $252 million in on‐location expenditures, $204 million in travel and lodging/housing, and $1.8 billion in fishing equipment purchases annually.^102^ Roughly a quarter of the total freshwater fishing−related spending comes from out‐of‐state anglers who are enjoying opportunities for fishing tourism. Nearly 11,000 jobs were supported by freshwater and recreational fisheries throughout the state.^103^ For Indigenous Peoples in the Great Lakes region and coastal Long Island, fishing is both a source of economic livelihood and vital to cultural traditions.

Of the approximately 20 million freshwater angler days reported on New York State waters in 2017, 44% were spent fishing for warmwater gamefish and 28% were spent fishing for coldwater gamefish. Anglers fished primarily on inland lakes and ponds (49%), inland streams and rivers (25%), and the Great Lakes and their tributaries (19%).^104^

The Ecosystems chapter of this assessment notes that warming waters adversely affect trout and other freshwater fish that have a relatively low thermal tolerance. Warmer lake and stream temperatures cause bodily stress, reducing activity and ultimately survival—and in the longer term, reducing the extent of suitable habitat for these coldwater species.^91^ Accordingly, the 2011 ClimAID report projected a shift in freshwater fishing in New York State from trout and other coldwater species to bass and other warmwater species.^75^ Total angler days for warmwater species rose from about 7.5 million days in 2007 to 8.8 million days in 2017 (23% increase), while coldwater fishing dropped from about 5.7 million to 5.4 million days over the same period (5% decrease).^104^, ^105^ Overall, angler days increased by 6% between 2007 and 2017, making the decrease in angler days for coldwater species even more noteworthy. Even more telling, perhaps, is the dramatic reduction in the number of angler days for rivers, which dropped from 6.5 to 5 million.^104^, ^105^

The 2011 ClimAID assessment called out the potentially significant impact of the shift from coldwater fishing to warmwater fishing on the economies and cultures of smaller, more rural counties where coldwater fishing is important—particularly in the Adirondacks.^75^ The case study in that assessment focused on three counties (Herkimer, Lewis, and St. Lawrence). Since ClimAID was published, all three counties have experienced reductions in revenue from spending on location by anglers who fished in those counties. Between 2007 and 2017, Herkimer and Lewis Counties experienced reductions of 36% and 33%, respectively, in the number of angler days.^104^, ^105^ These reductions suggest that climate change impacts are already being felt in this sector. Additional study is needed to assess future economic costs and potential adaptation options.

##### 3.2.2.3 Marine fisheries and seafood industry

Commercial marine fisheries (i.e., fishing operations that sell their catch for profit) and the seafood industry are the two primary industry sectors within New York State's marine economy. In 2020, these sectors generated roughly $6 billion in sales and supported more than 38,000 jobs.^106^

Ocean acidification, ocean warming, sea level rise, and increases in the frequency and intensity of storms are expected to have significant impacts on the marine fishing economy for both shellfish and finfish, affecting production, infrastructure, and sales, especially in marine aquaculture.^107^, ^108^ For shellfish aquaculture, increasing ocean water acidity reduces growth rates and size, the ability to grow shells, and overall survival rates. Acidification poses an especially serious risk to shellfish in early life stages.

As nor'easters and subtropical storms increase in intensity due to climate change,^40^ the potential for property damage also increases. Seaside operations and infrastructure are particularly vulnerable to wind, waves, and surging seas, brought on both by storms and by sea level rise. Seaside infrastructure that could sustain damage includes docks, buildings, refrigeration and ice‐making equipment, other processing equipment, hatcheries, and roads. Businesses can also suffer damage to or loss of gear and vessels. Lost days at sea and harvest area closures add to the potential for economic stress.

Research suggests that climate change may affect shellfish aquaculture more than finfish production.^109^ High air and water temperatures can harm productivity and raise food safety concerns due to increases in foodborne pathogens. Exposure to extreme heat, which is expected to increase with climate change, can also affect worker safety. Increases in the frequency, intensity, and duration of both storm and drought events can affect shellfish, especially shellfish aquaculture (discussed further in the Agriculture chapter).^62^ Heavy rainfall events, which have already become more common in the Northeast,^65^, ^110^, ^111^ harm marine shellfish aquaculture by introducing pollutants, excess nutrients, and other contaminants from surface runoff and stormwater. Shellfish are filter feeders and capture the pollutants in the stormwater, causing potential food safety concerns. Harvest operations are shut down following heavy rainfall events and only reopened once water quality improves. More severe storms could result in more harvest closures. The duration of such closures is also increasing as the size and duration of storm events increase.

Heavy rainfall and drought can also cause extreme fluctuations in seawater salinity. A high volume of freshwater runoff from a storm can temporarily reduce seawater salinity, especially near the coast where shellfish aquaculture is located. Drought, conversely, can cause increased salinity. Shellfish can tolerate a wide salinity range, but shellfish in earlier life stages can be vulnerable to extreme changes, especially low salinity. Higher salinity conditions create more favorable environments for certain shellfish parasites such as *Perkinsus marinus* (Dermo disease), a threat to both wild and farmed shellfish populations.^112^

#### 3.2.3 Finance, insurance, and investment

New York State is considered the financial and insurance capital of the United States. Many of the leading investment and commercial banks in the country are headquartered in the state, as are some of the top asset management, fintech, insurance, and reinsurance companies in the world. According to the New York State Department of Financial Services, the state is home to more than 1200 banking institutions, whose combined assets exceed $3.3 trillion, as well as more than 1700 insurance companies with combined assets totaling more than $5.5 trillion.^113^ The banking sector includes commercial banks, community banks, and credit unions, and is divided into retail, commercial, and investment banking. Services offered range from checking and savings to mortgages and asset management. While there are major regional banks headquartered in all parts of the state, employment in this sector is largely concentrated in New York City.

Despite the widespread presence of banking and credit institutions in New York State, it is important to recognize that access to formal banking and credit is uneven across different population groups, including LMI individuals, people of color, and new immigrants. Obtaining credit is particularly difficult for Indigenous Peoples due to the lack of formal private land ownership on Tribal lands.^114^ Access to insurance also varies across population groups. For LMI individuals, the affordability of hazard and property insurance is also a challenge.

According to the G20 Green Finance Study Group, the environmental and climate risks for the financial sector may be categorized as either physical or transition risks.^115^ Physical risk arises from the impact of extreme climatic events and environmental incidents. Transition risk arises from human efforts to address environmental and climate challenges.

The finance and insurance sectors are highly exposed to both physical and transition risks. Underwriting of property insurance policies is already being impacted by climate change, as property and casualty insurers hesitate to provide coverage of vulnerable assets in flood‐ and fire‐prone regions.^116^ Changes in the frequency and severity of climate events, such as hurricanes, floods, and wildfires, directly affect insurers’ liabilities, which threatens the long‐term viability of their business models. One analysis of risk exposure estimated that more than 834,000 single‐ and multi‐family residential homes in the New York City metropolitan area are at high risk of storm surges, with a total $334 billion reconstruction value.^117^ Thousands of commercial mortgage‐backed securities are at risk of losing value because the underlying properties are exposed to coastal flooding, including many that lie outside FEMA flood zones.^118^ Properties outside FEMA flood zones are more likely to be underinsured, a concern that may grow if flooding outside these zones becomes more frequent or severe. The possibility of stranded assets throughout New York State's coastal zones is a long‐term risk associated with sea level rise and permanent inundation by sea water.

Climate change poses many other material risks to the state's financial and insurance industries, where physical and transition risks resulting from climate hazards affect companies’ balance sheets—including both assets and liabilities.^119^ For example, climate‐related natural disasters may cause losses associated with business disruption and destruction of buildings and equipment, leading to an increase in disaster recovery costs and a reduction in revenue, potentially incentivizing business migration. In some regions of New York State, this may lead to lower property values, lower household and community wealth, and lower corporate profitability, translating into financial and credit market losses.^119^ To date, there has been a lack of accurate information and appropriate models to properly assess and manage the financial risks from climate change, and this (together with the lack of clarity in public policy) has affected the availability of capital to finance resilient infrastructure projects. However, a recent proposed rulemaking from the Securities and Exchange Commission on climate disclosures could create the space to assess these risks neutrally as part of standard prudent investment and portfolio management and could have a long‐term impact on future housing and real asset development.^120^

The impacts of transition‐risk drivers have already manifested themselves in the financial markets. For example, the cost of capital (the threshold of projected return needed to justify a project) for a new oil project has increased to around 20% for long‐cycle developments, while for renewable energy projects, it has dropped to between 3% and 5%.^121^ This has affected insurers as they are focused on improving pricing, determining risk, and matching premiums to values at risk for renewable energy projects. As the cost of capital goes down, and the costs of renewable energy projects and assets also continue to go down, there is a high risk of overinsuring projects. This would translate into higher‐than‐necessary premiums, unless policy limits are reconsidered, and would affect project sponsors; brokers; and procurement, engineering, and construction companies.^122^ For example, in 2020, a solar panel facility in Texas filed a claim of more than $70 million for damages caused by a violent hailstorm. This and other claims led insurance companies to reconsider the ways in which risks are assessed and used to determine insurance premiums.^123^ In the case of New York State, strong winds, wildfires, hailstorms, and lightning are considered the top climate‐related causes for material losses in renewable energy projects. Credit rating agencies are also considering material climate‐related risks in their assessments, but face significant challenges in adopting a more systematic approach to climate‐related risk integration.^124^

Climate change is already impacting financial stability and posing new challenges to financial regulators.^125^ In 2020, the G20 Financial Stability Board launched the Task Force for Climate‐Related Financial Disclosures with the objective of providing investors with recommendations for disclosing climate change risks in their portfolios. In 2019, the Network for Greening the Financial System recommended the use of climate stress tests to assess the financial stability implications of climate risks, eventually providing a series of scenarios that investors should consider in their climate financial risk assessments. Today, most New York‐based financial institutions include climate change in their assessments of financial risk, both internally and in relation to their portfolios’ resilience.

Climate change is also affecting financial resilience at the household and community level. As the frequency of extreme climate events continues to increase, highly exposed communities could experience significant property and infrastructure losses, which could result in reduced access to credit and an increase in the cost of home and personal insurance. In October 2021, the federal government's Financial Literacy and Education Commission, part of the U.S. Department of the Treasury, launched a nationwide study to explore climate‐related financial risks to households and communities, with specific emphasis on low‐income and historically overburdened communities.^126^ Another indirect climate impact that has the potential to affect entire communities is the possibility that insurers could decide to decline coverage altogether in some areas considered high risk for climate‐related events. Such a decision would directly affect valuations and tax structures in these communities. Several property insurers began to pull out of Long Island and other coastal areas in the early 2000s, citing increased risk from hurricanes and other storms.^127^ Their departure left former policyholders paying higher premiums for coverage from a special insurance program or from the “excess line” market.

In March 2023, the president of one of the largest reinsurance brokers (Aon PLC) warned the U.S. Senate Committee on the Budget that climate change is driving up prices and impacting the insurance industry, pushing companies to withdraw from “high‐risk areas, around wildfire and flood in particular.”^128^ In New York State, many regions are already experiencing an increase in policy premiums derived from past extreme climate events.^127^ However, as seen in states like Florida and California, insurance and reinsurance companies may choose to proactively exit high‐risk markets, where the ripple effects may disproportionately affect some populations in economically depressed regions.

While the assessment of financial climate risk is outside the scope of this report, it should be noted that climate risk has become a key factor in the design of shock scenarios and transmission channels and in understanding the conditions under which climate shocks can lead to amplified and persistent impacts on GDP.^125^ As a result, the limits of traditional approaches for the analysis of financial impacts of climate change and climate policies and their underlying assumptions are already being challenged by the uncertainty and path dependencies that characterize climate risks.

Incorporating climate‐related risk assessments into long‐term financial models could disproportionately impact LMI communities, communities of color, and immigrant communities. These communities already have limited access to credit and the financial resources needed to implement hazard mitigation programs, increase resilience, and develop capacity to adapt to climate change. Limitations on credit availability for manufactured housing could exacerbate challenges associated with climate event recovery in LMI communities. Because manufactured homes are considered personal property, they are not eligible for traditional mortgages and associated insurance.^129^ For Indigenous Peoples, credit access is a particular challenge due to the lack of private land ownership on Tribal lands.^114^ Private lending mechanisms often do not work well for Tribal Nations, potentially hindering their capacity to recover from extreme events.

### 3.3 Impacts on education

The education sector in New York State is made up of both public and private institutions, including pre‐K programs, K−12 schools, colleges and universities, and centers for life‐long learning (e.g., reskilling, language skills, adult education). The sector also includes educational programs such as those provided by Cornell Cooperative Extension, New York Sea Grant, and other entities that offer trainings, workshops, and other opportunities designed for youth and adult populations.

Most of the state's K−12 schools are in urban and suburban areas, where the state's population is concentrated. Within urban and suburban school districts, differences in educational quality and educational attainment have been documented.^23^ In many cases, these differences reflect community‐ and neighborhood‐level wealth inequalities. High‐performing, heavily resourced schools are more likely to be located in affluent suburban districts and high‐income urban areas, while low‐performing, poorly resourced schools are concentrated in low‐income communities, often urban neighborhoods with a higher proportion of people of color.

Rural districts also face challenges. Many rural districts contain only a single school where all K−12 students are educated. Many rural areas and rural school districts face acute financial resource pressures due to shrinking populations and local tax bases. At the same time, rural schools fill important roles in communities with few other civic organizations. They are often the designated emergency evacuation centers and provide a number of other, less tangible benefits to their communities.^130^

In addition to the vital work they do in educating the state's residents, public and private schools are a critical lifeline for the state's economy. Schools provide a safe environment for children during the day so that parents are able to work. A key lesson from the COVID‐19 pandemic was that the closure of schools, even when learning was shifted online, affected parents’ ability to work. This, in turn, contributed to reduced participation in the labor force, particularly among women.^131^

Climate change will affect many facets of the education sector in New York State. This section describes four critical areas of concern, all of which affect student performance and educational outcomes. These areas are impacts on K−12 schools, impacts on colleges and universities, impacts on school environments and infrastructure, and impacts on school budgets and costs. While this section focuses on the sectoral impacts of climate change, it is important to recognize that the education sector also plays a vital role in climate preparedness in New York State and in fostering just transitions, as discussed in Sections 5.2 and 6.1.2.

#### 3.3.1 Impacts on schools

The state's schools face a variety of climate‐related risks, including hurricanes, inland and coastal flooding, extreme heat, and extreme rain and snowfall events. These risks vary by region and depend on the location of a school. As shown in Figure 8‐7, flooding associated with coastal storm events and high tides, both of which are exacerbated by sea level rise, is a direct threat to schools located in coastal zones around New York City and Long Island. Hurricanes pose a substantial threat to schools in these regions (Figure 8‐8). Within New York City, approximately 200 school buildings suffered some damage as a result of Superstorm Sandy.^132^ After Hurricane Ida in 2021, approximately 17% of the city's school buildings were flooded, according to the New York City Department of Education.^133^

**FIGURE 8‐7:**
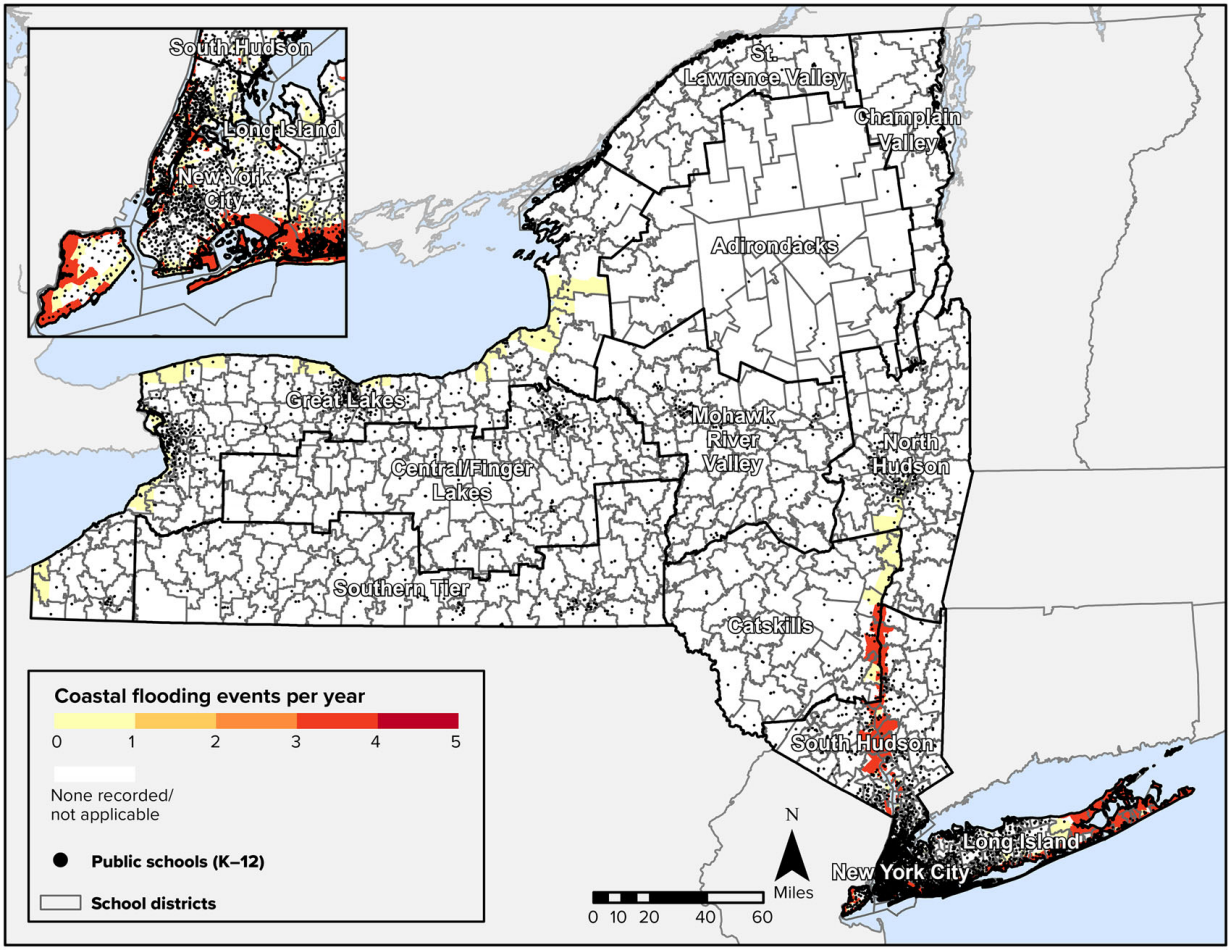
Locations of public schools in New York State, 2022, with average historical number of coastal flood events per year. Date ranges for the historical data used in calculating averages differ based on location and type of flood hazard present.^47^ Data from Federal Emergency Management Agency (2023)^42^ and New York State Education Department (2022).^135^

**FIGURE 8‐8:**
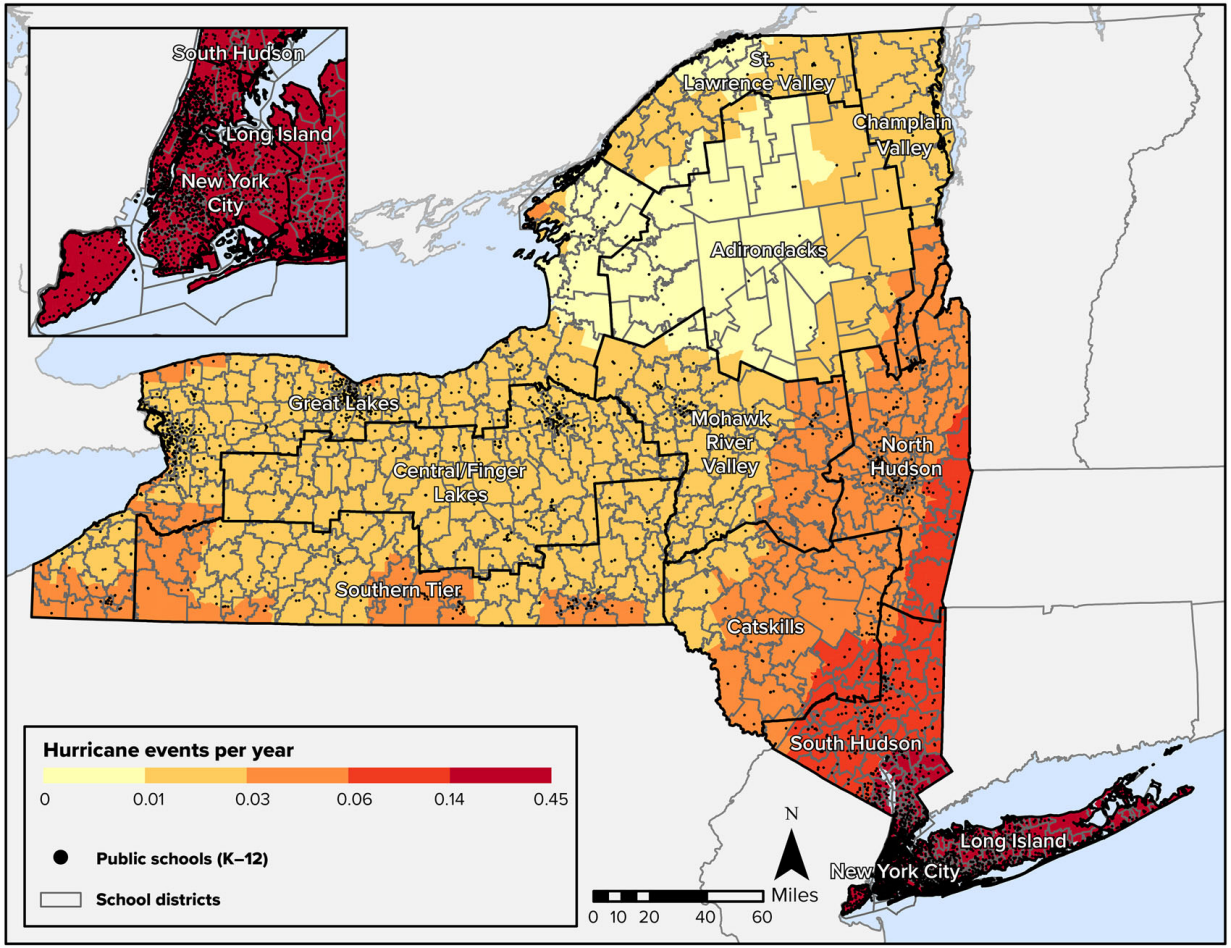
Locations of public schools in New York State, 2022, with average annual number of hurricane events, 1851–2020. Data from Federal Emergency Management Agency (2023)^42^ and New York State Education Department (2022).^135^

Schools located in flood‐prone inland regions may be threatened by increased flooding associated with more extreme precipitation events, as has been the case in other regions of the country.^134^ Increases in the severity of extreme snowfall events may also pose a hazard, especially in parts of the Great Lakes region, the Adirondacks, and the Southern Tier that already experience lake‐effect snow or high‐volume winter snowfall events (Figure 8‐9). Schools in these areas may be at risk from any added snow load on their roofs. A more detailed discussion of climate impacts on school buildings is provided in the Buildings chapter.

**FIGURE 8‐9:**
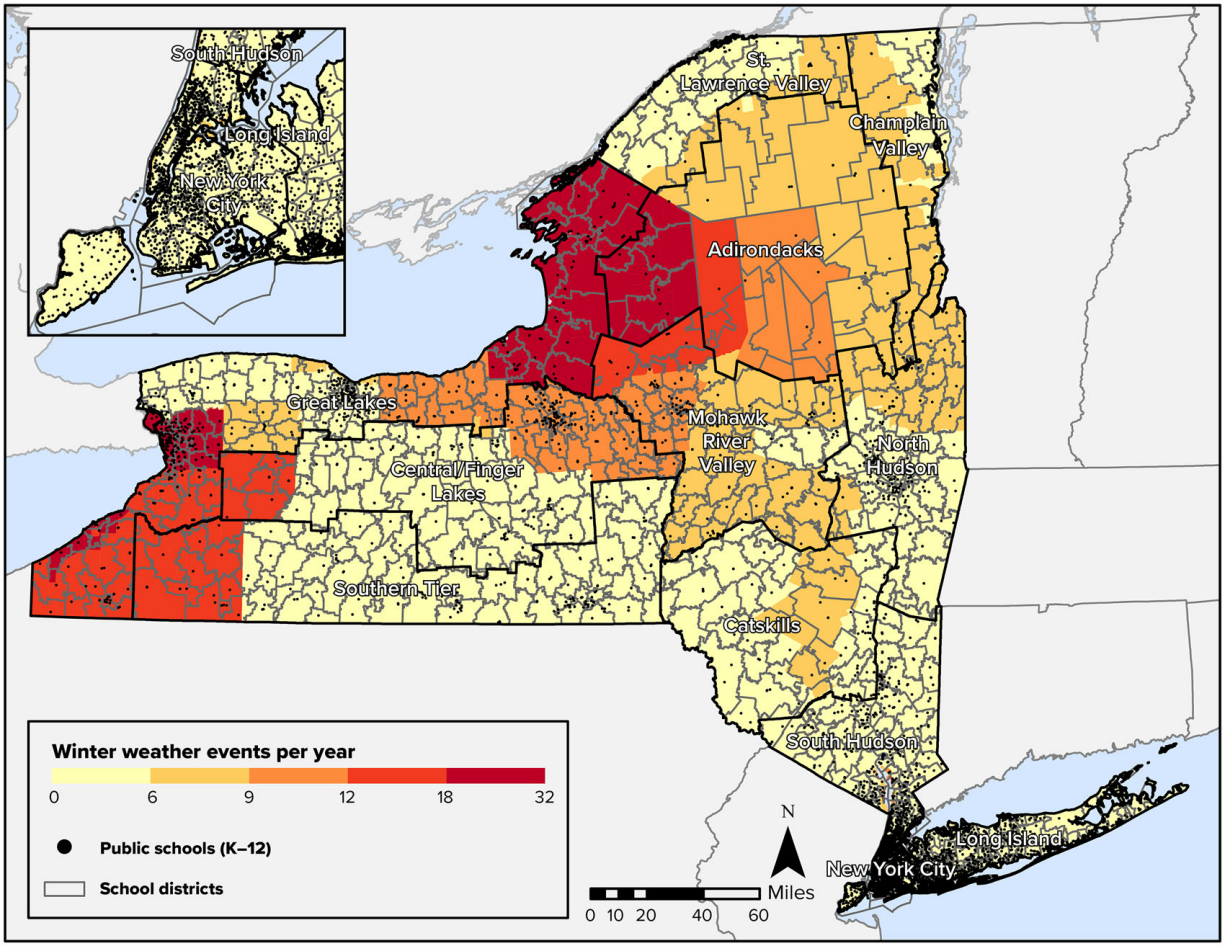
Locations of public schools in New York State, 2022, with average number of winter weather events per year, 2005–2022. Data from Federal Emergency Management Agency (2023)^42^ and New York State Education Department (2022).^135^

Rural districts with only one school may be particularly vulnerable if school is closed due to snow damage, flooding, or another climate disruption. In other states, the closure of schools because of extreme events has been found to directly affect school enrollments, student performance, and educational attainment and future income‐earning potential.^136^, ^137^, ^138^, ^139^ According to Noelle Short, district superintendent in Long Lake, the experience of the COVID‐19 pandemic has left schools in New York State better prepared to handle such emergencies (Short N, District Superintendent in Long Lake [2022, April 4, Personal communication]). For example, schools are better equipped to make timely transitions to online schooling in the case of a building closure. There is some evidence, however, that online schooling can have a significant negative impact on academic performance, especially for students from low‐income and single‐parent households. A national study of the impacts of school shutdowns during the pandemic found that students typically fell behind by 4 months in reading and 5 months in math.^140^ Students from low‐income households and historically overburdened communities fell even further behind. In math, students from households earning less than $25,000 a year fell behind by 7 months on average. Students attending majority‐Black or majority‐Hispanic schools fell behind in math by 6 months on average.^140^ Within New York City, school attendance rates in 2021 were below prepandemic levels,^141^ suggesting extensive educational losses for some students.

Closure of schools due to extreme events affects children from low‐income families in other ways, particularly those students who rely on free or reduced‐price school breakfast and lunch programs. Closure of schools has also been found to have an economic impact on households and families. A study of the economic impacts of flood‐related school closures in South Carolina estimated average economic losses of $215 per household per day.^142^

Recovery from all types of hazard events is more difficult for school districts that serve large proportions of students from socially vulnerable groups, as these students often face housing instability and food insecurity challenges and are especially vulnerable to the effects of disasters.^143^ Socially vulnerable students identified by the U.S. Government Accountability Office include students from low‐income households, racial and ethnic minorities, English learners, and students living with disabilities.

#### 3.3.2 Impacts on colleges and universities

Colleges and universities face additional challenges associated with climate impacts, including managing risks to student populations living in campus housing (also an issue for private residential schools). In 2021, flooding from the remnants of Hurricane Ida led to the evacuation of students from flooded residence halls at Stony Brook University.^144^ Low‐income college students, many of whom are already experiencing food and housing insecurities, face added risks associated with extreme weather, particularly if campus dining halls are closed and evacuation plans require them to vacate campus housing.^145^ For college and university students who live at home, extreme weather can disrupt student progress by exacerbating family or housing crises.

A changing climate may also affect university teaching and research facilities and programs. For example, flooding during Superstorm Sandy resulted in the loss of thousands of lab animals at New York University's Langone Medical Center.^146^ City University of New York (CUNY) campuses also experienced power outages and flooding during Sandy, and some CUNY facilities were used for sheltering evacuees from flooded areas of the city. As a result of these conditions, several CUNY campuses remained closed for nearly 3 weeks after the storm.^147^ Other universities in the New York metropolitan region, including Rutgers University in New Jersey, experienced power outages during Sandy that destroyed temperature‐controlled laboratory samples and led to loss of data for numerous scientific experiments.^145^

In response to Sandy and other recent extreme storm events, such as Hurricane Irene in 2011, many colleges in New York State have begun to develop climate preparedness plans. The plans include actions such as moving sensitive equipment out of basements, installing backup generators, adding air conditioning in dorm buildings, and developing emergency warning systems, communication protocols, and evacuation plans. Such plans can help ensure the safety of students, faculty, and staff and can also reduce climate‐related loss and damages.^145^

#### 3.3.3 Indoor school environments and student learning

Higher temperatures and humidity and increased flooding as a result of climate change may lead to problems with indoor environmental quality in schools—or exacerbate existing problems.^148^ Many schools throughout New York State are already experiencing a range of indoor environmental concerns, including visible mold and high levels of heat and humidity due to a lack of air conditioning and poor ventilation. These conditions have been shown to contribute to higher rates of student absenteeism in the state.^149^ Hotter school days have also been found to inhibit student learning, with especially large impacts on students of color.^150^ A study of New York City public schools found that student performance on standardized tests was 14% of one standard deviation lower when testing occurred on a hot day (90°F) as opposed to a more moderate day (72°F).^151^ Mold and poor air quality in schools can have an acute effect on students with asthma and other health conditions. Such children already miss more school than other children, with long‐term learning implications.^152^ Given the higher rates of asthma among low‐income communities and communities of color,^153^ any deterioration in indoor air quality in schools due to changing temperature patterns is likely to have a disproportionate impact on children in these communities. One solution is to install more air conditioning in schools, though this adaptation measure admittedly runs counter to efforts to reduce energy use and its associated greenhouse gas emissions.

In addition to its impacts on physical health, climate change is also affecting students’ mental health. Exposure to severe storm events, flooding, and other climate extremes has been found to have a direct effect on student mental health.^136^, ^154^ A study of the mental health impacts of Superstorm Sandy and Hurricane Harvey found that hurricane exposure can lead to increased symptoms of stress, anxiety, depression, and post‐traumatic stress disorder in people of all ages.^155^ Climate anxiety, a form of mental stress associated with concern and worry about climate change, is also a growing problem among all age groups, including the youth population, as discussed in Section 4.1.3.

#### 3.3.4 Impacts on educational costs and school finance

Climate change places additional financial pressures on public and private schools, colleges, and universities, as well as on state and local governments that are responsible for school funding. Rural schools in New York State already face financial pressures due to population decline. While extreme weather has not been documented as a cause of rural population decline and falling school enrollments in New York, this has been the case in other parts of the country. In North Carolina, for example, recent hurricanes have been found to contribute to population decline in rural areas with pre‐existing social and economic vulnerabilities, leading to decreased school funding and more school closures.^139^

Schools may also incur costs associated with increasing the resilience of school buildings to all types of climate hazards, including floods, heavier snow loads, and extreme rainfall and heat. Hotter school days may necessitate the installation of costly air conditioning systems, which would lead to higher energy usage and costs. While warmer winters may lead to reduced costs for winter heating, these savings may not offset the costs of installing and paying for increased cooling (refer to further discussion in the Buildings and Energy chapters). New York City invested more than $400 million to upgrade electrical systems and purchase air conditioners for classrooms between 2017 and 2022.^156^

Costs associated with school insurance may also increase in the future as damage due to climate hazards accumulates. The risk management department at New York Insurance Reciprocal (a school insurance company) reports that they “are still very much in the learning stages and trying to understand our vulnerabilities and how they may change/shift over time” (Rymarchyk G, Program Coordinator, Cornell Population Center, Cornell University [2022, February 3, Personal communication]). In all areas of New York State, there will be costs associated with the activities needed to monitor climate risks. These activities will include detailed and systematic audits of climate risks to schools and educational institutions, as well as monitoring of air quality and environmental conditions within schools.

Schools will also incur costs related to responding to school closures during extreme weather events. While overall snowfall in New York State is expected to decline, increases in the frequency of extreme snowfall events, ice storms, sleet, and freezing rain may nonetheless lead to more school closings, particularly in rural districts where students need to be bused long distances. Schools will face costs associated with continuing breakfast and lunch programs to ensure that low‐income students are not differentially impacted by school closures. Schools will also have to invest in the development of robust online educational delivery to ensure continuity of education following extreme events. One of the lessons of the COVID‐19 pandemic is that online schooling is possible as a response to climate disruption, but that there are associated costs. In terms of staffing, schools may need to hire additional social workers and other staff trained in mental health in order to address students’ mental health challenges. There will also be mental health and retention impacts on teachers at all levels as they struggle with the extra demands of shifting from in‐person to online instruction and back while also trying to meet the needs of students affected by the shifts.

Transition risk is also affecting schools and private academic institutions. In 2022, students at Ivy League universities filed legal complaints accusing the institutions of breaking the law by investing in fossil fuels. The complaints asserted that the universities were in violation of the Uniform Prudent Management of Institutional Funds Act, which according to the Climate Defense Project imposes a legal duty to put public interest first and to invest responsibly in future‐proof industries.^157^ In 2020, Cornell University, New York State's land‐grant university, voted to divest from fossil fuels, resulting in a moratorium on new private investments and bond vehicles focused on fossil fuels.^158^ By divesting, academic institutions may incur extra costs associated with compliance and the sale of the assets.

### 3.4 Impacts on culture, arts, and historic preservation

Museums, historic sites, and arts organizations are vitally important to the culture of New York State, and the significance of museums such as the Metropolitan Museum of Art and Morgan Library or sites such as the Statue of Liberty is easy to appreciate. Less well understood is the way local and regional museums contribute to their community's sense of place, understanding of a common history, and local pride. The state's many museums, historic sites, art galleries and arts centers, theaters, nature centers, zoos, and gardens attract tourists, employ locals and seasonal workers, and create opportunities for related service industries. Beyond the vibrant arts scene in New York City, other cities across the state provide homes for the arts as well, such as the Eastman School of Music in Rochester, which presents more than 700 concerts and recitals each year. Arts and cultural centers and organizations also enliven the state's small towns. For example, for more than 50 years, Blue Mountain Lake (population 143) has supported an arts center presenting performances, workshops, and exhibits. As discussed in this section, cultural resources in New York State are affected by climate change both directly and indirectly. Later sections (5.2 and 6.3) discuss the role of museums and cultural institutions in advancing adaptation and resilience.

#### 3.4.1 Museums, art centers, and historic sites

Much of New York State's history happened on waterways, and many of the institutions and historic sites that commemorate and interpret that history are vulnerable to flooding. Inundation caused by Superstorm Sandy in 2012, for example, destroyed the mechanical, heating, and electrical systems at the South Street Seaport Museum in Manhattan.^159^ During Hurricane Irene the year before, the Walter Elwood Museum in Amsterdam lost the collections on its first floor as well as its home, the 1774 Guy Park Manor, a state historic site (refer to the Small Museum Flooding Adaptations case study for more detail). Examples of endangered historic sites include the Montauk Lighthouse on Long Island and the Statue of Liberty, both vulnerable to coastal erosion.^160^, ^161^ The Shinnecock Nation Cultural Center and Museum is the first and only museum on Long Island that is owned and operated by Native Americans and dedicated to honoring their ancestral and living history. This museum, which serves nearly 10,000 visitors every year, is at constant risk of flooding, which could result in the loss of artifacts, documents, and other materials related to Shinnecock and Eastern Woodland history and culture.^162^ This flooding will become even more of a threat as climate‐related extreme weather events continue to affect coastal communities in New York. Coastal archaeological sites are also vulnerable to coastal erosion and could be lost entirely.^163^

Climate‐related threats to museums and historic houses and sites go beyond flooding and structural damage. Loss of electricity can lead to loss of climate control in a museum's collections areas. Excessive or prolonged humidity can damage collections, and rapid changes in humidity and temperature are both harmful to collections as well. All cultural institutions depend heavily on income from visits, performances, and programs. If they close because of damage or uninhabitable conditions, they lose essential revenue. In the longer term, higher temperatures will increase the load on, and the cost of, climate control in museums, which is essential to maintaining collections.

Cultural organizations can face difficulties in preparing for, and recovering from, climate change−induced damage. Large institutions in major population centers often have better access to resources than small, rural ones. After Superstorm Sandy hit Manhattan, the Mayor's Office of Resiliency, in collaboration with the New York City Economic Development Corporation, included South Street Seaport in its Financial District and Seaport Climate Resilience Master Plan “to protect lower Manhattan from the urgent threat of climate change.”^164^ By contrast, a small foundation that owns and interprets the Penfield Pond Dam, part of a historic district in Ironville where electricity was first used in an industrial application, has no such resources. The dam, originally constructed in the late 1820s to power Taft and Penfield's ironworks, was compromised during Hurricane Irene in 2011. The state government has ordered the small Penfield Foundation to remove the dam. The cost—more than $1 million—is far more than the group can afford. In addition, the removal of the dam would be a loss to historic interpretation at the site and to the many people who enjoy recreating on and around the pond it impounds. A further danger to the foundation is that it would be liable for damages downstream if the dam failed. The foundation has not been able to obtain assistance from federal or state sources.^165^

#### 3.4.2 Cultural impacts and loss of cultural ecosystem services

The state's cultural heritage extends beyond institutions and relates to both tangible and intangible cultural legacies, as well as the diversity of values and community identities.^166^ Researchers, practitioners, and policymakers have begun to recognize the cultural dimensions of climate change impacts.^167^ While climate change is sometimes portrayed as a threat to cultures and ways of life, it is important to recognize that cultures are not fixed but are actively and dynamically learned and shared, and cultural change is likely to be a critical component of climate change adaptation.^38^

Some of the most important cultural impacts of climate change in New York State are likely to be related to loss of or changes in cultural ecosystem services. While some benefits associated with ecosystem services are easily quantifiable (e.g., tourism, covered in Section 3.2), cultural ecosystem services are the nonmaterial benefits people find in nature,^168^ which are often difficult to quantify. Researchers are finding ways to quantify these intangible, cultural benefits in ecosystems, such as viewsheds and scenic corridors. For example, one project applied ecosystem services characteristics and valuation criteria from various global, national, and localized studies to Cazenovia Lake in New York State, proposing a framework for quantifying these cultural services and methods for visual resource management.^169^ Other noncultural ecosystem services provided by the environment, such as carbon sequestration, should be noted^170^ and are discussed elsewhere (Ecosystems chapter).

In the region that is now New York State, the cultures of the earliest human inhabitants were tied to the environment. The Haudenosaunee are an example of one such people. One source described the Haudenosaunee as “linked socially, physical [*sic*], spiritually, and culturally to the environment, the Natural World. The links are numerous, reciprocal, and depend on each being in the Natural World living one's life according to the Haudenosaunee traditional teachings. This requires specific acts and beliefs between people and the rest of the Natural World for there to be peace and justice and continuity of the culture.”^171^ The Haudenosaunee and the other Indigenous Peoples of the state have struggled to preserve their cultures in the face of environmental change ever since the first Euro‐American settlers arrived in the early 17th century. Climate change is one more environmental stressor, but it promises to change the interconnectedness of all parts of Indigenous cultures much faster and more drastically than the dams on the St. Lawrence, the industrial pollution of Oneida Lake, or the wheat farming in Central New York during the early 19th century.

Climate change will have numerous cultural and livelihood impacts on Tribal Nations and Indigenous Peoples. These impacts may include loss of habitat for traditional practices such as gathering wild foods, fishing, and trapping.^172^ They also may include reduced production of maple syrup and apples,^62^ as well as loss of the ash trees that have traditionally provided materials for basket weaving in some cultures.^91^ Indigenous sports and music traditions may also be affected by climate change. The sport of lacrosse, for example, relies on wood from ash trees for the fashioning of traditional sticks.^173^, ^174^ While impacts on Indigenous music have not been studied in New York State, one study suggests that climate change may affect Indigenous musical cultures as a result of impacts on natural materials used to construct instruments.^175^ In considering the impacts on Tribal and Indigenous cultures, it is also vital to recognize links between the natural world and traditional practices, and the cultural beliefs that are interwoven with them.^176^ Although Indigenous cultural traditions are particularly vulnerable to the effects of climate change, traditional knowledge is also helping Tribal Nations understand and cope with the changing environment, as discussed in Section 4.2.1.

Many other cultural practices of both native and non‐native inhabitants of the region are also deeply tied to the environment. As noted in Section 3.2, a changing climate will affect many outdoor activities that are particularly important in the rural parts of the state, such as hunting, fishing, and birding. Winter activities such as undeveloped skiing (i.e., telemark, cross‐country), snowshoeing, ice hockey, ice fishing, and snowmobiling are already seeing the effects of climate change. Coastal and inland recreational areas of the state are also venues for important cultural traditions, providing gathering spaces for families and friends to celebrate summer holidays including Memorial Day, Juneteenth, Independence Day, and Labor Day, as well as numerous local winter festivals and carnivals. As noted in Section 3.2, these spaces and associated activities are threatened by climate change−related changes such as HABs and warmer winter seasons with reduced snowfall.

### 3.5 Impacts on government and civil society

Government agencies have critical roles, functions, and responsibilities that support healthy and viable communities and economies. In New York, the state and local governments collaborate in the development and implementation of climate adaptation, hazard mitigation, and disaster recovery plans. At times, the cooperation between state and local agencies extends to designing and implementing local policies pertaining to zoning, environmental review, public health, and climate action.

#### 3.5.1 State government and climate change planning and response

The Office of the Governor, the legislature, state departments, and most government agencies play a central role in implementing and funding climate change planning and preparedness initiatives in New York State. Several state government authorities, agencies, departments, and commissions provide essential services and information to residents, local governments, schools, and nonprofit organizations to develop and implement climate change mitigation and adaptation projects and programs. They do so by providing direct services to New Yorkers and directing resources to help local governments and school districts develop responsive adaptive capacity, implement adaptation and resilience projects, and develop and implement climate change adaptation and resilience plans and local hazard mitigation plans.

For example, the Department of Environmental Conservation's Office of Climate Change provides guidance, funding, and technical assistance to develop local climate change adaptation and resilience plans and funds the implementation of climate adaptation and resilience projects through Climate Smart Communities grants.^177^ Other examples include the Division of Homeland Security and Emergency Services, which manages FEMA's disaster mitigation programs and serves as the state's primary hazard mitigation planning and emergency response and disaster recovery agency, and the Disaster Preparedness Commission, which includes a total of 31 state agencies and the American Red Cross and oversees the preparation of the state's disaster plans, including state and federal government coordination.^178^

The availability of federal funds, as well as dedicated state funds such as the Environmental Protection Fund and the Clean Water, Clean Air, and Green Jobs Environmental Bond Act, is critical to helping the state government enhance economic and climate resilience. For example, New York State added the climate change mitigation and adaptation account to the Environmental Protection Fund in fiscal year 2016−2017, and $24 million was appropriated for climate change projects in the first year.^179^ The Environmental Bond Act was approved in November 2022, authorizing the spending of $4.2 billion, including $1.1 billion for restoration and flood risk reduction, $650 million for water quality improvement and resilient infrastructure, and $650 million for open space land conservation and recreation.^180^ During the COVID‐19 pandemic, the federal government provided $12.75 billion in financial assistance to New York State through the State and Local Fiscal Recovery Funds under the American Rescue Plan Act of 2021.^181^ In addition, the state relied on other sources of American Rescue Plan Act funds for emergency rental assistance, home energy assistance, childcare relief, and other services.

New York State agencies and authorities are participating in climate change vulnerability assessments and subsequent climate change adaptation plans. According to the draft assessments, New York State agencies and authorities are already experiencing a variety of climate change impacts that are disrupting their ability to conduct normal operations, including delivering services and programs (Lowery MD, Assistant Director, New York State Department of Environmental Conservation, Office of Climate Change [2021, March 16, Personal communication]). Climate‐related impacts are interfering with state employees’ ability to deliver services, travel to and work safely at state offices and field sites, and maintain state‐owned and state‐managed constructed assets, including office and incarceration facilities and infrastructure and natural assets like parks, forests, and wildlife management units. Climate change impacts are expected to increase disruptions to agency assets, operations, and missions, as well as revenue generated from user fees and service delivery. For state government agencies, climate change acts as a threat multiplier by adding stresses to existing risks and problems; it can also increase costs to agency operations and repairs after extreme weather events. There may also be an increase in demand for state agencies’ guidance, regulations, funding, and services to help New Yorkers (including special needs populations, local governments, businesses, schools, nonprofit service providers, and other entities) adapt to and respond to climate change.

#### 3.5.2 Local governments and civil society

State and local governments experience climate impacts and engage in climate action in different ways.^182^ Local governments frequently rely on community‐ and faith‐based organizations to bolster their capacity to respond to climate events and disasters.^183^ These organizations represent key stakeholders supporting and demanding local government actions to prepare for climate change. Just like government entities, they must develop the necessary capacity to anticipate and adapt to the impacts of climate change. Local governments also collaborate with counties and with other nonprofit entities, frequently relying on these organizations to reinforce their responsive capacity to address climate impacts.

When local governments experience disruptions in their internal operations, tax revenues, and ability to offer basic services, the state government can redirect resources and programs to help local governments develop responsive capacity, create local climate change adaptation and resilience plans, and implement resilience and hazard mitigation measures. To support local governments, New York's state government provides financial aid, technical assistance, and coordination among agencies, including post‐disaster coordination through several state agencies, including the Department of Environmental Conservation's Office of Climate Change, the Department of Homeland Security and Emergency Services, and the Office of Resilient Homes and Communities. State agencies also provide programs like Climate Smart Communities, which helps local governments take action to reduce greenhouse gas emissions and adapt to a changing climate.

One study investigated the capacity of local governments in New York State to address climate change through both adaptation and greenhouse gas reduction.^184^ It found that urban municipalities have much greater participation in climate action than rural municipalities and that key barriers to action include small size and associated lack of human, financial, and technical resource capacity.

Following the economic shock of the COVID‐19 pandemic, local governments have experienced climate change as a threat multiplier, disproportionately affecting vulnerable population groups. As described in the Energy chapter, climate change is altering patterns of energy demand through increasing temperatures and prolonged periods of extreme heat or cold.^185^ Some cities have experienced periods of unsustainable energy demand and power outages that affect many communities, including densely populated LMI neighborhoods. In response, local governments have sometimes deployed emergency financial and technical resources to provide temporary solutions such as warming centers and facilities for people to get dry ice and water.^186^ Further discussion of the impacts of power outages can be found in the Buildings chapter.

Services offered by local governments can also be disrupted by other facets of climate change, including out‐of‐season weather events such as late winter storms, fall heat waves, and uncharacteristically dry summers. These events can lead local governments to alter annual budgets and comprehensive plans, as well as municipal services such as public works, community services, and recreation services.

The impacts of climate change have led some municipalities to place a new priority on hazard mitigation and climate action planning, aimed at increasing physical and economic resilience. Section 5 discusses the role of climate adaptation and resilience planning as a key component of the climate adaptation process. In addition, the need to address climate change is affecting the ways in which cities undertake other short‐ and long‐term planning and development. As climate conditions change, zoning and environmental reviews will need to be updated on an ongoing basis to reflect the latest understanding of hazards, risks, and vulnerabilities, including anticipatory approaches to address uncertainties. According to the Department of Environmental Conservation, “New York's State Environmental Quality Review Act requires all state and local government agencies to consider environmental impacts equally with social and economic factors during discretionary decision‐making.”^187^ In the context of climate change, environmental reviews may be used to limit future development in floodplains.

Climate change has also had an impact on community participation in local government. For example, some municipalities have observed a drop in participation in town hall meetings during unseasonably cold or hot days (City and township leaders, New York State [2023, March, Personal communication]). At the same time, concern about climate change has led to the creation of community‐based organizations focused on disseminating information and creating knowledge networks to increase climate and economic resilience. Climate‐focused organized advocacy groups are also increasingly active in local and statewide public consultations, legislative sessions, and rate cases.

#### 3.5.3 Crime, fraud, and price gouging

Heat waves, floods, and other extreme events are often perceived as triggers for criminal activity. The perception that property crime increases following a disaster may be partially rooted in racial biases among the public and in bias in how crimes are reported in the media. In particular, there is often a heightened focus on petty crimes of residents of color.^188^ However, little research has been conducted on this topic in New York State. By contrast, a much more serious, but less visible, crime that is common after disaster events is fraud. Types of fraud include insurance fraud, scams involving fraudulent charitable organizations, and fraudulent repair efforts.^189^

Other related crimes include misuse of recovery funds designated for adaptation and price gouging following an extreme event. In the aftermath of Hurricane Henri in 2021, for example, the New York Attorney General issued a consumer alert warning both consumers and businesses to watch for potential price gouging on essential items and services. New York State's price gouging law bans companies from taking advantage of a crisis to charge excessive prices for vital and necessary goods and services. In March 2022, the Attorney General launched a rulemaking process to fight “price gouging and corporate greed,” following reports that major corporations were using the COVID‐19 pandemic and inflation as an excuse to unfairly raise the prices of basic goods.^190^

Given the magnitude of resources directed to New York State for disaster relief, and the potential for abuse in the form of fraud and price gouging, this is an area that requires continued attention.

#### 3.5.4 Fiscal impacts on local governments and financial institutions

The economic and fiscal impacts of climate change are adding to the pressure felt by local governments already suffering from the effects of the COVID‐19 pandemic. The compounding effects result in long‐term economic and fiscal shocks that negatively affect tax revenue as at‐risk industries leave local jurisdictions and relocate to less vulnerable regions, leaving behind unemployment, brownfields, and stranded assets, disproportionately affecting vulnerable population groups. Because municipalities depend upon land‐based finances, rising sea levels may also result in reduced municipal property tax revenue in coastal municipalities that experience inundation of low‐lying properties.^191^, ^192^ Reductions in tax revenue impact local governments’ ability to fund infrastructure projects, obtain adequate flood insurance, and issue debt (i.e., borrow funds to pay back later), which is necessary to invest in increasing climate and economic resilience. When combined with the costs associated with climate adaptation, including the development of climate action plans and the implementation of hazard mitigation actions such as avoided development and home buyouts, this decrease in tax revenue has the potential to affect local governments’ fiscal stance.

State and local governments rely on banks and insurance companies for debt services to fund transportation, education, and health infrastructure. In 2022, the state‐related debt in New York was $61.9 billion, which represented a 5.5% increase compared with 2021.^67^, ^193^ Credit rating agencies for local governments focus on the current state of the local economy to determine municipal creditworthiness. In evaluating a local economy, agencies look at insurance premiums, threats to the tax base, fiscal strategies, and climate risk reduction actions taken by the municipality, among other factors. Specifically, rating agencies investigate how climate vulnerability affects, and will affect, state and local governments. For example, according to Moody's Investor Services: “by 2040, rising sea levels and greater risk of frequent flooding will affect most states’ coastal counties, including more than 110 cities with a population greater than 50,000. Economic weakening, higher maintenance costs and lost tax revenue are particular credit risks for state and local governments over the next several decades.”^194^ The prospect of credit deterioration due to climate change has already altered bond investors’ perceptions of risk. As a result, local governments issuing authority bonds and municipal bonds have had to adopt new climate risk disclosure standards to ensure a more accurate risk assessment.^120^ As a result of heightened risks, the costs of bonding will also increase.^195^ This will be particularly relevant as some local governments in New York State begin to see a decrease in tax revenue due to population changes.^196^

As of 2018, local governments in New York State (excluding New York City) registered a total of $43.6 billion in debt.^197^ Going forward, local governments will have to consider climate risks as they continue their reliance on debt instruments to fund infrastructure projects or to supplement flood insurance. When climate risks are not addressed, or when there is a determination that climate change may directly impact the government's debt service coverage ratio or its ability to provide basic services, credit ratings can be downgraded, affecting the government's ability to issue debt. In the future, climate impact and recovery planning may become a requirement for local governments, mandating the identification of stakeholders, vulnerable populations, and climate disaster response agencies.

## 4 VULNERABILITY, EQUITY, AND ENVIRONMENTAL JUSTICE

This section considers the vulnerabilities of population groups, communities, places, and businesses and workers to the climate risks and impacts described in Section 3. The concept of vulnerability emphasizes economic, political, and social factors that make particular regions, communities, groups, and individuals more sensitive to harm from climate change and less able to cope and respond.^38^ Vulnerability to climate change is nearly always intertwined with other factors that contribute to inequalities, including differences in assets and access to economic, social, and political capital.^30^, ^198^, ^199^ For many groups, particularly Indigenous Peoples and members of racial and ethnic minorities, climate vulnerabilities are also rooted in legacies of dispossession, displacement, discrimination, and redlining.^200^, ^201^, ^202^ Moreover, many factors influencing vulnerability are intersectional, such that particular groups often face multiple forms of disadvantage due to factors such as income status, race or ethnicity, age, gender or sexual identity, and limited English proficiency.^198^

While the focus of this section is on vulnerabilities, it is important to recognize the leadership roles that members of many historically marginalized groups and communities have played in climate justice and related climate movements. Youth, LGBTQ+, environmental justice, and Tribal and Indigenous communities, among others, are making vital leadership contributions to climate movements and climate action in New York State, as well as nationally and globally.

### 4.1 Vulnerable populations

Several broad categories of people within New York State's population are vulnerable to climate change impacts, including LMI individuals and households, older adults, youth, immigrants, the unhoused, and those who identify as LGBTQ+. There is a distinction between vulnerable populations and vulnerable communities (refer to Section 4.2), where the former refers to population groups that share a common attribute (e.g., age, socioeconomic status) that distinguishes them from the rest of the population, and the latter refers to communities that have been historically marginalized due to Indigeneity, race, or ethnicity. For both categories—population groups and marginalized communities—vulnerability is often exacerbated by intersections of multiple forms of disadvantage, as well as by discrimination. Many of those facing intersectional climate vulnerabilities also faced disproportionate health and economic effects during the COVID‐19 pandemic. In some cases, the pandemic pushed already‐vulnerable individuals into new realms of vulnerability, expanding the ranks of people who have dropped out of school, live paycheck to paycheck, or are unable to cover rent or mortgage payments. The pandemic has also created a new vulnerable group: those living with the chronic effects of post‐COVID conditions.^203^

#### 4.1.1 Poverty and LMI populations

Poverty is a significant intersectional factor that shapes exposure to climate risks. People who fall below the poverty line have limited housing options, as well as limited capacity to prepare for and respond to extreme events. Food insecurity and energy insecurity add to their vulnerability. Importantly, poverty status often intersects with other factors that shape climate vulnerabilities, such as Indigeneity, race, ethnicity, gender identity, age, housing status, and LGBTQ+ status. As shown in Figures 8‐10 and 8‐11, household income and poverty rates are highly variable across New York State. Despite having a higher average income and higher wage levels than the state overall (refer to Section 2.1.2), New York City also has higher rates of poverty. In 2021, 18% of the city's population lived in poverty, whereas the rate was 13.9% for the state overall.^204^ Broken out by race and ethnicity, the poverty rate was 20.3% among people identifying as Black, 20.9% for people identifying as Hispanic or Latino, and 9.6% for people identifying as non‐Hispanic or Latino white.^204^

**FIGURE 8‐10:**
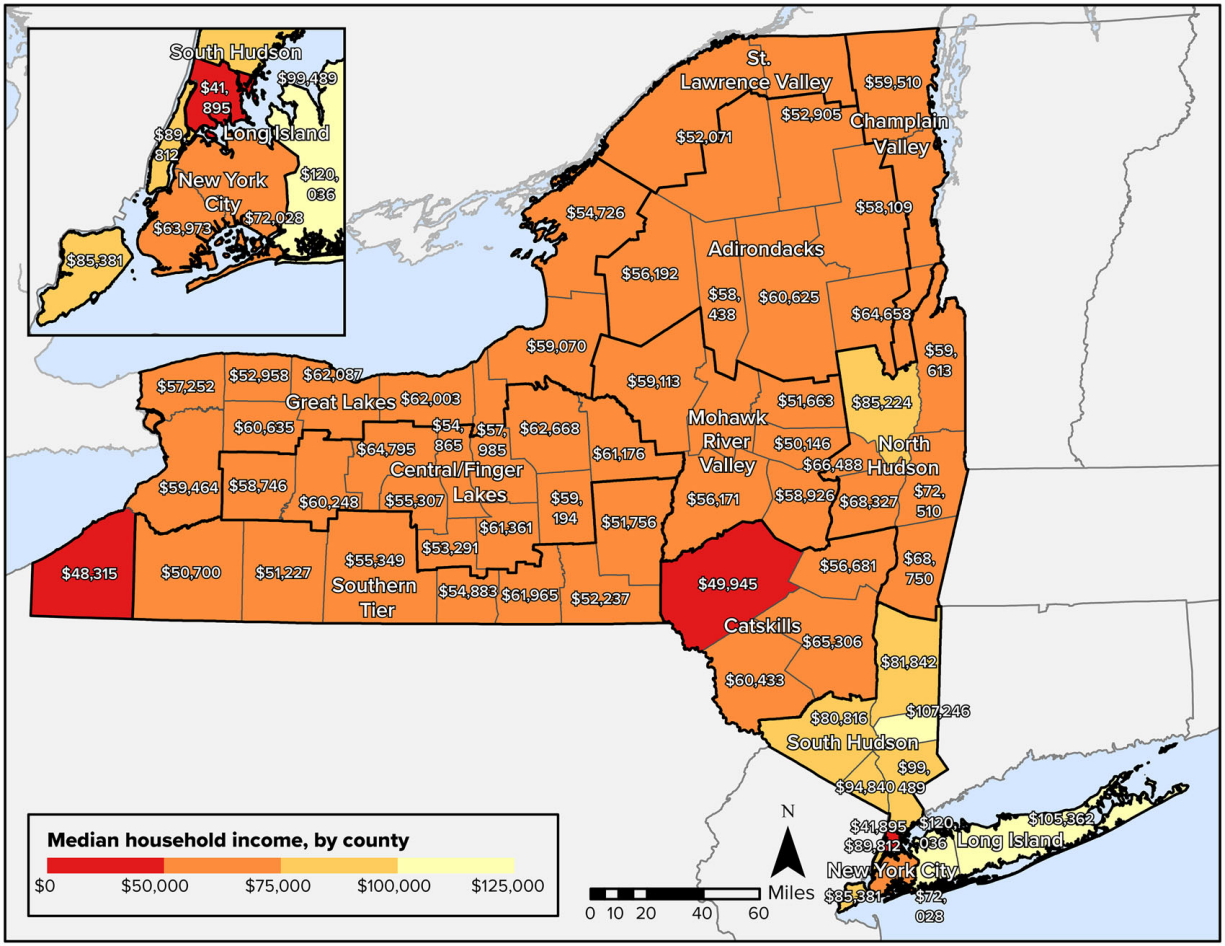
Median household income by county for New York State, 2016–2020. Data from U.S. Census Bureau (2020).^205^

**FIGURE 8‐11:**
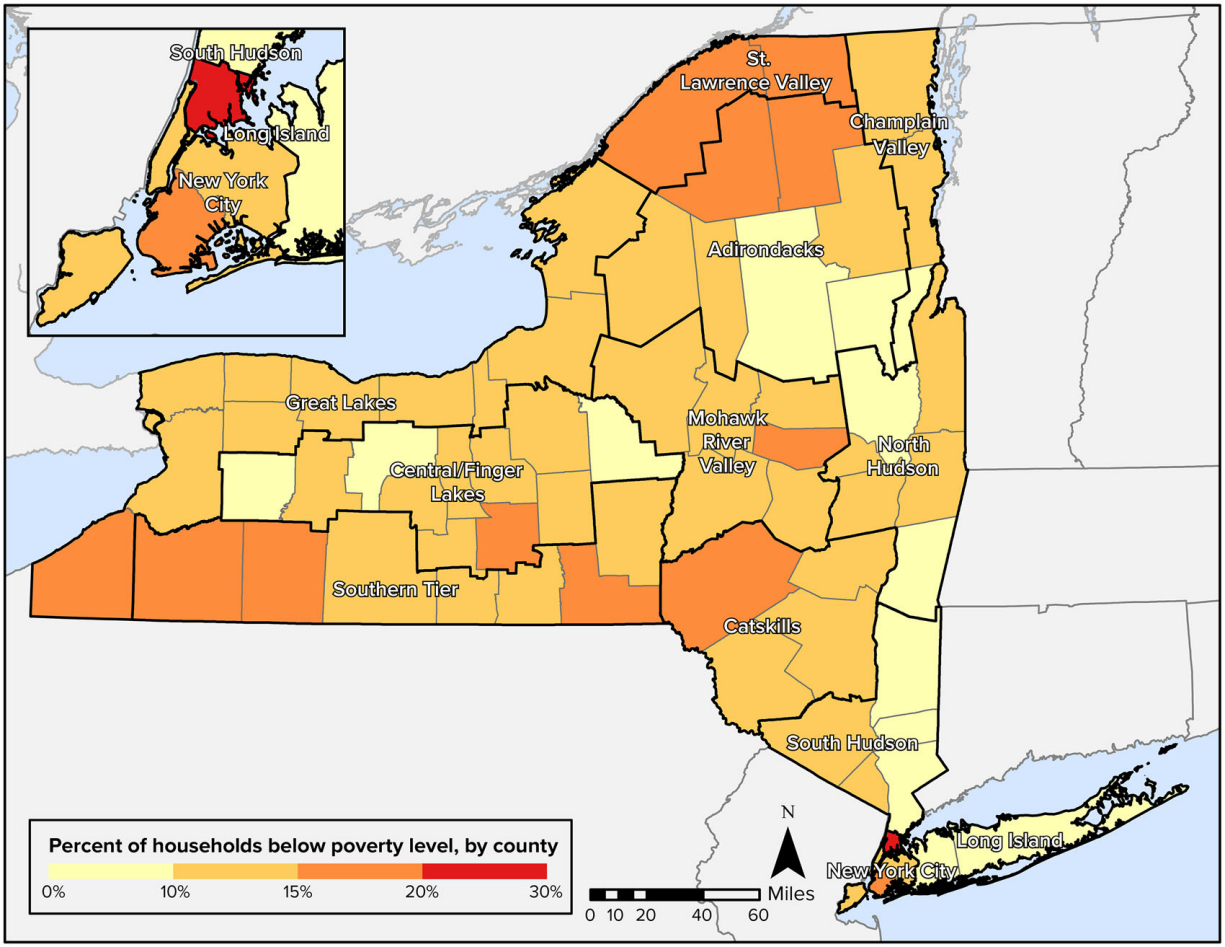
Percentage of households below poverty level by county for New York State, 2016–2020. Data from U.S. Census Bureau (2020).^206^

Like households below the poverty line, other LMI households in New York State also face added climate risks. According to the U.S. Department of Housing and Urban Development (HUD), low‐income populations include individuals whose income is up to 50% of the median income in the area in which they live. Moderate‐income individuals are those whose income is between 50% and 80% of the area's median income.^207^ LMI populations may be vulnerable to the impacts of climate change for several reasons. Many LMI families in New York State are living paycheck to paycheck. Climate change−related disruptions that lead to short‐ or long‐term business closures can be economically devastating for them. LMI status also underlies many factors that contribute to vulnerability, such as energy, food, and housing insecurity. For example, energy insecurity associated with LMI status may increase the likelihood of exposure to extreme summer heat due to limited ability to afford air conditioning. An LMI family that is already food insecure will be further harmed by rising food prices caused by climate‐related disruptions of food production.^62^ LMI individuals and families who are housing insecure or lack flood and property insurance may be more likely to live in flood‐ or hazard‐prone areas, may have limited ability to evacuate during extreme storm or flood events, and may not have the assets needed to recover from such events.

BOX 2 **Low‐ to moderate‐income populations**

HUD presumes that people from a number of population segments are low‐ to moderate‐income, including abused children, battered women, older adults, severely disabled adults, unhoused persons, illiterate adults, persons living with AIDS, and migrant farm workers.^207^ These individuals face particular challenges in society and may have difficulty gaining employment, staying housed, and accessing a number of other essential services. Other groups that can be added to this definition include undocumented immigrants and immigrants with limited English proficiency who, as a result, face barriers to securing stable housing and living wage employment.

#### 4.1.2 Populations of older adults

As mentioned in Section 2.1, New York State's population is aging.^208^ Statewide, approximately 3.5 million or 17.5% of people are 65 or older, which is higher than the national average of 16.8%.^209^ That number is expected to increase and reach at least 4.5 million by 2040, accounting for 22% of the state's population, and as much as 25% in certain regions.^61^

Climate change poses particular threats to older adults and to communities that include an unusually high percentage of older adults. Older individuals are more vulnerable to climate hazards for a variety of health‐related reasons. For example, as people age, their ability to thermoregulate decreases, and they have a reduced tolerance for extreme heat and cold. Older workers are at greater risk from heat stress, particularly in sectors that require outdoor work. Older individuals who have physical mobility challenges also face added risk associated with extreme events such as flooding, where evacuation may be difficult or impossible. (Refer to the Human Health and Safety chapter for a fuller discussion of health vulnerabilities associated with age and underlying health conditions.)

Older adults, along with low‐income New Yorkers, were among those most impacted by Superstorm Sandy.^210^ In the aftermath of the hurricane, many older individuals were trapped in upper‐floor apartments for a week or longer due to power outages and associated lack of elevator service.^211^ Of those in New York City who died as a result of Sandy, close to half were over 65 years old.^212^

A number of factors make older adults especially vulnerable to climate change impacts. Older LMI individuals who are living on a fixed income may already be experiencing housing, energy, food, and health insecurities, all of which could be exacerbated by climate change. These challenges are further compounded by living arrangements. Social isolation and aging, both on the rise,^213^ intersect to make individuals especially vulnerable to the impacts associated with climate change.^214^ For example, studies show that older adults who live alone are at greater risk of dying during a heat wave.^215^ New research also shows that older immigrants are more likely to experience loneliness than older nonimmigrants.^216^

Trends toward aging‐in‐place can also add to the vulnerabilities of older adults, particularly for mobility‐limited individuals who may face difficulties with evacuation during extreme storm events. Both New York State and many communities within the state have joined the AARP's Age‐Friendly Network to prepare communities for more aging‐in‐place in the future. Incorporation of early warning systems and storm preparation plans into such networks is an important step for enhancing resilience in communities with growing shares of older residents. Rural communities that are aging at a faster‐than‐average rate tend to have local economies with smaller working‐age populations and a smaller base for local taxes, which fund many weather‐related services.

#### 4.1.3 Youth populations

For children and young people, climate change poses both an immediate and a long‐term threat to their physical and mental health and well‐being.^217^, ^218^ Very young children are especially susceptible to harm from extreme events and from extreme heat and cold.^153^ For older children and teens, the mental health impacts of climate change may be associated with direct experiences of extreme events. These experiences can contribute to depression, anxiety, and post‐traumatic stress disorder, all of which can impede healthy physical, social, and emotional development.^38^ Young people are also increasingly subject to climate anxiety and depression, including feelings of grief, sadness, and hopelessness about the future.^219^

However, young people are not simply passive victims of climate change. Youth climate change activism is growing worldwide.^220^, ^221^ Recent climate marches in New York City were partly organized by youth.^222^ The effort to save Hangar Theatre in Ithaca, following a devasting flood, was organized in part by the youth‐based Sunrise Movement. People who work with youth on climate change find that they are fired with enthusiasm and determination to make a difference, although they have not, so far, been brought fully into the conversation.^221^, ^223^

Young people will be more impacted by climate change in their lifetimes than today's older generations, and their experiences of climate change are very different.^38^ As discussed in Section 5.2, educational systems are critical for preparing young people for an emerging mid‐21st‐century climate‐changed world that is very different from today's world.

#### 4.1.4 Immigrant populations

New immigrants to New York State are another group that could have heightened vulnerability. Reasons for their vulnerability could include higher‐than‐average rates of poverty, limited English skills, anti‐immigrant racism, and other factors. In addition, many immigrants live in the state without legal immigration status. Approximately 16% of both eligible‐to‐naturalize immigrants and undocumented immigrants report incomes below the poverty line, compared with 13.9% for the state as a whole.^224^, ^225^ Linguistic isolation is also present in both groups: among those eligible to naturalize, 13.2% report speaking no English and another 22.8% report speaking a little English. These rates are 10.2% and 27.4%, respectively, among undocumented immigrants. Limited English skills could hinder the ability of some immigrants to understand storm warnings and disaster alerts, particularly in areas of the state where such alerts are only in English. Another broad measure of the potential vulnerability of immigrant populations is their health insurance status: 90% of eligible‐to‐naturalize immigrants have health insurance, while fewer than 60% of undocumented immigrants do.^224^ Undocumented immigrants are also more likely to be engaged in natural resource, construction, or maintenance occupations, where exposure to heat and cold is common. Such workers have few legal protections and may not be able to complain about such exposure and related unsafe working conditions.

Another factor that could contribute to the vulnerability of new immigrant populations is a lack of knowledge about the locations of local flood risks, particularly for those living in rental housing. In comparison, longer‐term residents are more likely to have had prior experiences with flooding. Housing discrimination against immigrants, and difficulty obtaining legal housing for undocumented individuals, may also increase the likelihood that immigrants will live in flood‐exposed or otherwise unsafe housing. Of the 13 victims who drowned in New York City due to the extreme rainfall from Hurricane Ida in September 2021, 11 were immigrants living in illegal basement apartments.^226^ (Refer to the Hurricane Ida Vulnerabilities in New York City case study for further discussion).

New immigrants also typically have difficulty accessing government resources following disaster events. Undocumented status has historically resulted in the exclusion of immigrants from receiving emergency cash assistance and disaster recovery assistance from the federal government. During the COVID‐19 pandemic, for example, undocumented immigrants were not eligible for federal stimulus checks and enhanced unemployment benefits. As a result of advocacy from immigrant rights organizations and community‐based groups, New York State created an Excluded Workers Fund to help meet the basic needs of undocumented New Yorkers who would otherwise have been shut out from receiving emergency federal cash assistance. As this example suggests, New York State's actions are in part necessitated by federal government policies that shift burdens to states. New York State's Office of New Americans helps immigrant populations access information and promotes transparency about their rights to government resources.

#### 4.1.5 Unhoused and housing insecure populations

More than 90,000 individuals in New York State are estimated to be unhoused on any given day.^227^, ^228^ The majority of these people live in New York City, but other cities including Buffalo, Rochester, and Syracuse also have large numbers of unhoused individuals,^229^, ^230^, ^231^, ^232^ and unhoused individuals also live in every county in the state.^233^ Many unhoused individuals have significant underlying health problems, and a lack of safe and secure housing significantly exacerbates exposure to climate hazards and shocks, including heat waves, periods of extreme cold, heavy rainfall and snow events, and flooding. Some cities in New York State activate emergency weather safety plans to provide food, shelter, and transportation to unhoused individuals during extreme events, such as the December 2022 blizzard that dropped 50 inches of snow on Buffalo. Despite Buffalo's “Code Blue” safety plan, several unhoused individuals died in that storm.^234^, ^235^ In addition, news reports have identified climate‐related housing displacement as a potential contributor to homelessness.^236^

Beyond the unique vulnerabilities of the unhoused, LMI New Yorkers also face growing pressures from rising rents and housing shortages, particularly shortages of affordable housing. Home prices and rents have increased dramatically throughout the state in recent years. While rising rents and concerns about housing affordability in New York City have received much attention in the media,^237^ every county in the state has seen its median home price increase since 2020.^238^ In many rural counties, including Schuyler, Hamilton, Sullivan, Herkimer, and Ulster, median prices climbed 20% or more between 2020 and 2022.^238^ As discussed in the Hurricane Ida Vulnerabilities in New York City case study, rising rents and lack of housing have meant that many LMI households are living in illegal conversions, including basements and garages, which are especially vulnerable during extreme precipitation and flood events.

In the future, if climate migration increases and intersects with remote work opportunities, housing shortages and lack of affordable housing could become growing stressors, affecting planning and development in smaller urban areas that are already have limited infrastructure and social services. There is a clear need to increase the supply of permanent affordable housing in New York State. Beyond that, there is also a pressing need to improve how low‐income and other vulnerable households displaced by extreme storm events are temporarily housed and transitioned into permanent housing, especially considering an overburdened shelter system that is highly difficult to access and navigate for housing‐insecure families. This need is especially pressing for immigrant families with limited English proficiency (refer to Section 4.2.3).

#### 4.1.6 LGBTQ+ populations

LGBTQ+ populations within New York State have been significantly affected by persistent discrimination, the HIV/AIDS epidemic, violence, and cultural erasure. It has been estimated that, by 1995, 1 in 15 gay men nationwide and 1 in 10 within the male 25−44 age cohort had died as a result of the HIV/AIDS epidemic, a group sometimes referred to as the “lost generation” or “missing generation.”^239^ Without census data regarding sexual orientation and gender identity, it is challenging for demographers and other social scientists to evaluate the impacts of climate change on LGBTQ+ populations. Nonetheless, higher concentrations of LGBTQ+ people in a number of coastal cities and states may indicate unique vulnerabilities and challenges not experienced by other segments of the population.^240^ For example, LGBTQ+ individuals, particularly LGBTQ+ youth, are more likely to be unhoused than other groups,^241^ contributing to greater exposure to heat, cold, and extreme weather events.

The LGBTQ+ community has historically faced uneven challenges during the distribution of aid following a natural disaster. During Hurricane Katrina, because of the disproportionate number of unhoused youth self‐identified as LGBTQ+ and the lack of legal recognition of LGBTQ+ families, FEMA was unable to provide the same level of support it gave to the rest of the affected populations.^242^

#### 4.1.7 Undercounted populations

Undercounting of population contributes to climate‐related risks for particular population groups. According to the U.S. Census Bureau, the 2020 Census undercounted Black, Hispanic, and Native American residents, mostly in populations concentrated in cities and Tribal areas.^243^ Undercounting poses a risk to these groups as it may affect representation, climate adaptation plans, and allocation of city services such as hazard mitigation for the next 10 years.

According to the U.S. Census Bureau's estimates, the 2020 Census missed counting 5 out of every 100 Hispanic residents, 5.6 out of every 100 Native American residents, and 3.3 out of every 100 Black residents at the national level. While young people were also undercounted, and older people were overcounted, this was in line with previous censuses.^243^ Some of the reasons for undercounting, including language barriers, literacy rates, lack of internet access, and distrust of the federal government, may be the same reasons why these population groups are at higher risk of being disproportionately affected by climate change. Undercounting can also drive divestment and divert federal and state resources from programs like Justice40 and from initiatives under the Climate Leadership and Community Protection Act (the Climate Act). Specifically, it can impact funding for weatherization, fortifying the electric grid and sewer systems, cleanup of Superfund sites, flood mitigation, and fire risk prevention programs. Undercounting can also affect the way communities prepare for and respond to climate events.

### 4.2 Vulnerable communities

For many communities and groups in New York State, disproportionate vulnerabilities to climate change are rooted in the state's colonial, imperial, and racist history. This history dates back to the displacement of Indigenous Peoples and includes actions such as the razing of the African American community of Seneca Village in Central Park in 1857,^244^ the redlining of communities of color from the 1930s to the 1960s,^245^ the racial profiling of African Americans and other minorities by police,^246^ the rise in anti‐Asian racism, and other ongoing forms of discrimination. This subsection highlights the vulnerabilities of such communities, paying particular attention to groups affected by the legacies of discrimination and ongoing systemic and structural racism, including Tribal Nations and Indigenous Peoples, communities with environmental justice concerns, and LMI communities of color.

#### 4.2.1 Tribal Nations and Indigenous Peoples and communities

Like other Indigenous Peoples throughout North America, the Tribal Nations located in New York State have experienced centuries of territorial dispossession that have resulted in the loss of lands and threatened their access to vital natural resources and sacred sites.^35^ Polluting industries have historically been located on Indigenous lands, and toxic pollutants contaminate sites such as Onondaga Lake, referred to by the Haudenosaunee (Iroquois) as Where the Water Meets the Willows.^247^ Tribal communities and Indigenous Peoples also face many social challenges that contribute to vulnerability, including high rates of poverty and low levels of educational attainment.^114^, ^248^

Climate change adds to the challenges these communities face.^50^, ^202^, ^249^ It also threatens their cultural traditions. Great Lakes Indigenous Peoples face threats to cultural traditions like ice fishing. Members of the Shinnecock Indian Nation face threats to shellfish harvesting along the Atlantic coast.^250^, ^251^

Numerous sites throughout the state have social, cultural, historic, archaeological, or educational importance to Indigenous Peoples. For example, across Long Island, there are many sites that are significant to the Shinnecock Indian Nation. To ensure that those sites remain undisturbed to the maximum extent practicable, the town of Southampton uses a cultural resource protection overlay district.^252^ Climate change poses new or added risks to many of these sites, including threats to land due to sea level rise.^250^

While documentation of climate‐related risks to Indigenous communities is critical, it is also vital to recognize and acknowledge the long history of survival and resilience among Indigenous Peoples and Tribal Nations in New York State. As discussed by Robin Wall Kimmerer, Tribal populations within the United States were historically relocated to areas with different climatic conditions from their homelands and were forced to learn how to grow food and survive in different climates. Kimmerer—a citizen of Potawatomi Nation from Wisconsin, which was relocated to Oklahoma—suggests that displacement contributed to the resilience of Tribal Nations via learned experience and sharing of knowledge.^176^, ^253^ As discussed in Section 5, many Tribal Nations have developed detailed climate change adaptation plans that offer additional insights and lessons on resilience.

#### 4.2.2 Environmental justice communities and LMI communities of color

The U.S. Environmental Protection Agency defines environmental justice as “the fair treatment and meaningful involvement of all people regardless of race, color, national origin, or income, with respect to the development, implementation, and enforcement of environmental laws, regulations, and policies.”^254^ Environmental justice communities include communities that are disproportionately exposed to environmental pollutants associated with industrial production, transportation, energy production, sewage treatment, and similar activities.^255^ Often LMI communities of color, environmental justice communities have experienced decades of private disinvestment and a lack of meaningful government support. Such communities are more likely than others to be redlined, designated as blighted and targeted for slum clearance or urban renewal, or designated as HUD entitlement communities. Climate‐related stressors can overlap with existing forms of disadvantage and environmental harms experienced in many environmental justice communities.^256^ Compounding these double exposures to climate and social stressors, residents of these communities also often lack financial resources such as flood insurance to aid in recovery from extreme events such as floods and coastal storms.

In some environmental justice and LMI urban communities of color, there are growing concerns about population displacement. Displacement can occur due to climate hazards, such as more frequent flooding, but also as a result of climate resilience projects that contribute to gentrification pressures. This process, sometimes described as “climate gentrification,” happens when green infrastructure measures and other climate resilience projects such as enhanced flood control contribute to higher property values in environmental justice and other LMI communities.^257^, ^258^, ^259^ Climate gentrification has been documented in cities such as Philadelphia, Miami, and New Orleans.^257^, ^259^, ^260^ Another potential contributor to climate‐related displacement in these communities is the promotion of urban neighborhoods as “climate friendly” as a way to attract new residents.^258^ Displacement of residents can fragment or fracture social networks and lead to loss of social and economic capital. This, in turn, can exacerbate the vulnerability of environmental justice and LMI communities to extreme climate events and related hazards. To ensure that adaptation efforts do not contribute to displacement, it is critical that efforts to enhance urban climate resilience and attract new residents reflect the needs and values of local environmental justice and LMI communities of color. These and other issues are explored in more detail in the Adaptation and Equitable Site Remediation case study.

#### 4.2.3 “Disadvantaged” communities

As part of the work taking place under the Climate Act, New York State recently undertook a large‐scale effort to identify and map the state's disadvantaged communities. (Note that the term “disadvantaged” is codified in the Climate Act.) This effort, led by the Climate Justice Working Group, developed a set of criteria for identifying disadvantaged communities in order to ensure that frontline and underserved communities benefit from green energy transitions, associated reductions in air pollution, and economic opportunities.^261^ According to these criteria, a community is considered disadvantaged based on a combination of environmental and climate risk burdens, demographic characteristics associated with climate vulnerability, and health vulnerabilities.^261^ These communities sometimes overlap with the communities discussed in Sections 4.2.1 and 4.2.2, but not always. As illustrated in Figure 8‐12, disadvantaged communities can be found in all assessment regions except the Adirondacks. High concentrations of disadvantaged communities occur in both urban and rural areas, including the New York City, South Hudson, Catskills, and Great Lakes assessment regions. In each of these areas, more than one‐third of census tracts are designated as disadvantaged (refer to Table 8‐3).

**FIGURE 8‐12:**
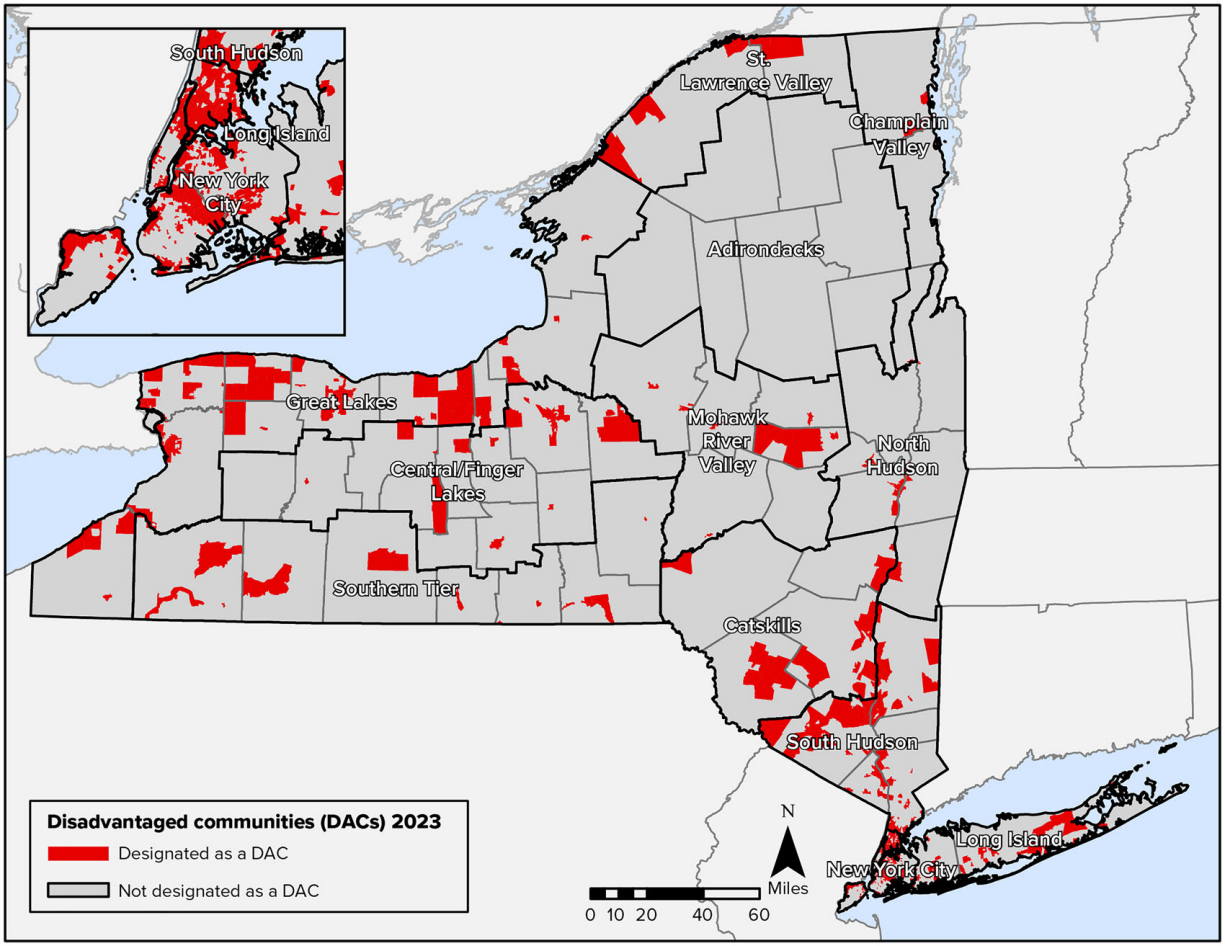
Disadvantaged communities in New York State, 2023, as identified by the Climate Justice Working Group. Data from New York State Energy Research and Development Authority (2023).^262^

**TABLE 8‐3:** Percentage of census tracts within each assessment region designated as disadvantaged communities.

Region	Percentage of tracts identified as disadvantaged communities^a^
Adirondacks	0%
Catskills	38%
Central/Finger Lakes	27%
Champlain Valley	12%
Great Lakes	37%
Long Island	14%
Mohawk River Valley	26%
New York City	44%
North Hudson	22%
South Hudson	42%
Southern Tier	26%
St. Lawrence Valley	25%
**Total**	**35%**

*Note*: Data from New York State Energy Research and Development Authority (2023).^262^

^a^ Here, the “disadvantaged” designation is as statutorily prescribed by the Climate Act.

### 4.3 Vulnerable places and locations

This section examines vulnerability through a spatial lens. It discusses vulnerabilities common to communities located in particular types of places (e.g., rural areas) or in specific regions (e.g., the Great Lakes region). It explores some of the factors that increase the risk of climate change impacts in these locations. This section is not intended as a comprehensive review of all vulnerable locations in New York State. Other types of location‐based vulnerabilities, such as urban heat vulnerabilities, are discussed in other chapters of the assessment.

#### 4.3.1 Rural areas

The state's rural areas display unique vulnerabilities to climate change. Rural economies that depend highly on climate‐sensitive industries such as tourism and natural resources have heightened vulnerability due to their limited economic base.^263^ The COVID‐19 pandemic exacerbated this vulnerability, as counties throughout New York experienced decreases in leisure and hospitality sector employment,^264^ consistent with widespread anecdotal reports of business closures and labor shortages. Rural economies that depend on agriculture are vulnerable to climate‐related disruption of crop or livestock production.^62^, ^265^ Rural communities are also prime candidates for double exposure, whereby other social and economic stressors such as endemic poverty, poor health, and drug dependency overlap with climate‐related stressors to exacerbate vulnerabilities.^266^

The population profile of rural areas adds to their vulnerability. Rural areas of the state tend to have a higher proportion of older residents, many of whom are living on limited or fixed incomes. The aging and shrinking of populations in rural communities leads to reduced tax bases, which in turn affect the capacity of these areas to prepare for, respond to, and recover from extreme climate events.^265^ Among local governments, rural towns are among those with the most limited capacities for developing and implementing climate resilience and preparedness plans. As discussed in Sections 3.3 and 3.4, rural schools, museums, and arts institutions also face added adaptation challenges due to isolation, small size, and limited financial resources.

#### 4.3.2 Great Lakes communities

The sub‐basins of the Great Lakes—which include the lakes themselves along with associated water bodies such as the Niagara and St. Lawrence Rivers and Lake Champlain—have experienced changes in water levels over time, with recent wide shifts. For example, Lake Ontario moved from lower‐than‐average levels in 2012 to record highs in 2017. Projections of future lake levels are inconclusive, meaning it is uncertain whether lake levels will increase or decrease overall.^40^ However, climate models generally agree that annual and multi‐year variability in Great Lakes water levels will increase, leading wide shifts to become more common, driven by periods of drought and extreme precipitation events.^40^

These changes pose a challenge to communities in the region, including many small, rural municipalities and Indigenous communities. Persistent, extreme low water levels can negatively affect Great Lakes communities in a variety of ways, including impacts on shipping and navigation, water quality, and wetland health. By contrast, extremely high water levels can result in flooding along Great Lakes shorelines, as occurred in 2017 on Lake Ontario.^267^ Such flooding can last for months at a time, compromising infrastructure, limiting and in some cases preventing access to homes, and eroding shorelines at accelerated rates. In addition, extreme fluctuations between high and low water levels over short periods of time can affect water quality, contribute to erosion, affect public and private infrastructure and public health, and limit or prevent access to water resources and waterfront properties.^268^ Further discussion on this topic can be found in the Village of Sodus Point Water Level Adaptations case study.

The experiences of lakeside communities in the Great Lakes, including recent catastrophic flooding events along Lake Ontario, reinforce the need to incorporate local perspectives and knowledge in resilience planning. These experiences also point to a need to enhance communities’ technical capacity and access to credible resources, tools, and data to prepare for a changing climate. In response to these needs, a number of efforts have been initiated to develop coordinated resilience networks. Two such programs that have provided funding and planning to support resilience efforts in the region are REDI (Resiliency and Economic Development Initiative) and CLEAR (Coastal Lakeshore Economy and Resiliency). Through REDI, the state government has committed up to $300 million to benefit communities and improve resilience in flood‐prone regions along Lake Ontario and the St. Lawrence River.^269^ CLEAR supports local communities in the same regions with the development of long‐term comprehensive resilience strategies. By partnering with communities and private institutions, CLEAR relies on local knowledge and perspectives to assess different long‐term scenarios and the potential impacts and opportunities within each region. The program's work has produced a series of recommendations for local action, such as updated zoning, and helped lakeshore communities and water‐dependent businesses develop capacity to increase resilience.^270^

#### 4.3.3 Atlantic coastal communities

Atlantic coastal communities in New York State are facing special challenges, including growing threats from sea level rise and storm surge.^271^ These communities, which include portions of New York City^272^ and the south shore of Long Island,^273^ vary widely in terms of socioeconomic characteristics. Some Atlantic coastal communities are wealthy, especially those on the far eastern end of Long Island. Others have large concentrations of LMI households. Many waterfront neighborhoods in New York City (e.g., Brooklyn, the Rockaways) are low‐income communities with large populations of non‐white individuals, immigrants, and older adults and may thus be considered frontline communities.^46^ Among these communities, many families live in public housing developments that are highly vulnerable to storm surge, as was seen during Superstorm Sandy.^274^

Other issues faced by Atlantic coastal communities in the state include limited ingress and egress pathways that make it difficult for first responders to access areas during flood and storm surge events; bridges that may not be rated highly enough to allow for the weight of emergency vehicles; and transportation infrastructure that fails to function during power outages. Breezy Point in Queens, New York City, is one such coastal community. Entry and exit from the community is limited to one flood‐prone road, utilities lack redundancy and faced power disruptions during Superstorm Sandy, and the bridges serving the peninsula were closed in advance of Superstorm Sandy.^275^ As highlighted in Section 4.2.1, the Shinnecock Indian Nation on Long Island is another Atlantic coastal community that faces unique climate‐related challenges, including loss of land due to sea level rise.

Some Atlantic coastal communities already experience significant localized “sunny day” flooding due to their low elevations and high tides. In addition to economic disruption, this flooding can also affect the emotional health and well‐being of residents. Researchers have documented the social and emotional impacts of living with tidal flooding in Jamaica Bay, Queens.^276^ Their study also showed that local communities are critical sources of adaptation knowledge and use collaborative adaptive social networks for communication, cooperation, and support.

Further compounding the risks from sea level rise and “sunny day” flooding are heavy precipitation events, which are expected to increase in frequency in the Northeast. Many Atlantic coastal communities have high levels of impervious surfaces, and many also have combined sewer drainage systems with limited capacity to manage an increase in stormwater runoff. During heavy precipitation events, combined sewer systems may be pushed beyond their capacities and overflow into local waterways, resulting in discharges of untreated sewage. Storm surge may carry that untreated sewage back into those communities through household plumbing fixtures and manholes, as observed during Superstorm Sandy.^277^ This type of flooding presents substantial health risks, as residents may be exposed to bacteria normally associated with sewage, toxins from various chemicals, mosquito‐borne infectious diseases, and infectious agents such as leptospirosis (spread largely through rat urine). Such flooding events are especially damaging to the health and well‐being of frontline communities, and particularly LMI renters and homeowners, who have limited financial resources for recovery and rebuilding.^278^ For many immigrant communities on the front lines of climate change, the lack of FEMA assistance for noncitizens is particularly challenging. Superstorm Sandy was also devastating for many middle‐income homeowners who discovered they had insufficient flood insurance to cover the full costs of repair and rebuilding.^278^

Flood‐prone inland communities on Long Island, in New York City, and in the South Hudson assessment region face challenges similar to those of coastal communities.^40^ Instead of storm surge, they may be susceptible to riverine flooding caused by overbank flooding, flash flooding, dam and levee failure, and other events.^279^ For further discussion of the hazards, risks, and vulnerabilities faced by many Atlantic coastal and flood‐prone inland communities, refer to the Resilient Homes and Communities Programs (formerly the New York Rising Community Reconstruction Program community plans).^280^

### 4.4 Vulnerable businesses and workers

This section focuses on businesses and workers that are disproportionately affected by the impacts of climate change. As discussed in Section 3, economic sectors such as outdoor tourism, natural resources, timber, and fisheries are among those most vulnerable to income and employment disruptions associated with the impacts of a changing climate. This section highlights businesses that face added climate change vulnerabilities because of small size and access to resources. It also discusses the vulnerabilities of different types of workers. Vulnerabilities of workers in sectors such as Agriculture and Transportation are covered elsewhere in the assessment.

#### 4.4.1 Small and minority‐owned businesses

As of 2018, 98% of the businesses in New York State had fewer than 100 employees, and small businesses accounted for more than 54% of employment.^281^ Small businesses rely on access to urban streets, roads, and highways; affordable and reliable broadband internet; reliable energy, water, sewer, and waste services to support operations; reliable and timely supply chains; safe and accessible storefronts and office space; availability of reliable, healthy, and productive employees; and access to customers. Small businesses, particularly minority‐owned small businesses, are more vulnerable than other businesses to extreme weather events for many reasons. For example, they may have lower cash reserves and greater difficulty in obtaining rebuilding loans. They may have limited capacities for developing and implementing climate preparedness plans, and those renting space may lack flood and property insurance.^282^, ^283^ Small businesses and minority‐owned businesses are also more vulnerable because they are less likely to have relationships with mainstream banks and to have commercial leases or subleases. Following disaster events, small businesses and minority‐owned businesses are less likely to reopen than larger businesses.^284^, ^285^ One study of the business impacts of Superstorm Sandy found that business closures were concentrated among small, stand‐alone establishments.^284^ Similarly, a study of the business impacts of Hurricane Katrina found that minority‐, women‐, and veteran‐owned businesses were more likely than other businesses to close permanently after a disaster event.^285^

Disruptions to the functioning of small businesses not only lead to loss of income but can have cascading impacts on the local community, especially if a business plays a critical role in the community.^286^ For example, closures of small‐ and medium‐sized businesses eliminated the main sources of food for local residents of the Rockaway Peninsula of New York City in the wake of Superstorm Sandy, when the region was isolated from the rest of the city due to storm damage.^277^ The closures also contributed to income and job losses, adding further obstacles to obtaining food and reducing resources available for recovery.^277^ Within the Rockaways, local residents cited permanent closures of businesses in the community after Superstorm Sandy as a significant barrier to recovery.^277^

#### 4.4.2 Frontline, outdoor, and public service workers

Vulnerabilities to climate shocks vary among different types of workers. The experience of COVID‐19, for example, revealed substantial differences in impacts between workers in the information and knowledge sectors, where work could be done remotely, and workers in service and retail industries, where in‐person work was required. For workers in retail, hotels, restaurants, personal services, and related sectors, COVID‐19 shutdowns led to reduced hours, job losses, employment uncertainty, and reductions in wages.^287^ Within New York State and throughout the United States, these impacts fell disproportionately on Black and Hispanic workers, and especially female workers, who make up a larger‐than‐proportionate share of workers in these sectors.^287^

Superstorm Sandy showed a similar pattern in its differential impacts on workers. The storm caused a loss of nearly 30,000 jobs in New York City, with especially substantial impacts in the restaurant and food service sectors in Lower Manhattan.^288^ Temporary shutdowns of businesses following Sandy had a disproportionate negative impact on hourly, frontline service workers in the retail, tourism, and recreation industries. All of these industries employ a disproportionate share of women.^289^ Unlike salaried workers, who continued to be paid, many hourly wage workers went unpaid when businesses shut down.^64^

Climate change is also a health threat to outdoor workers in all sectors, including bike delivery workers, construction and utility service workers, and agricultural workers, as well as public service workers such as police, firefighters, and emergency responders. All experience direct exposure to extreme weather events such as heat waves, extreme precipitation, high wind events, and wildfires. Many of these workers are also critical for disaster response and recovery, rebuilding, and repair. As discussed in the Agriculture chapter, agricultural workers, particularly migrant workers, may face overlapping stressors due to extreme heat and increased use of agricultural herbicides and pesticides in response to crop threats.^62^

While direct estimates of the impact of climate change on labor productivity in New York State are not yet available, recent aggregate global estimates suggest that rising temperatures and humidity are already having a significant negative impact on labor productivity worldwide.^290^ One study estimates that more than 650 billion hours were lost annually during the period of 2001−2020, compared with a 1981−2000 baseline, due to increased heat and humidity.^291^ Using a measure of labor effectiveness (a combined measure of hours worked and labor productivity), another study estimates that labor effectiveness in the Americas for those working in full sunlight may decrease by an average of 5.6% under a scenario of 3.6°F (2°C) of global warming.^292^ Additional estimates of the impacts of climate change on labor productivity in New York State are currently underway in a separate report.

## 5 ADVANCING ADAPTATION AND RESILIENCE

Throughout this chapter, the discussions of climate impacts and vulnerable populations have included numerous examples of adaptation measures being used in New York State to either moderate the negative effects of climate change or move communities, businesses, and other institutions toward resilience. Some of these examples are summarized in Table 8‐4. Due to the breadth of the society and economy sector, which covers everything from government and the economy to education and the arts, it is not within the scope of this chapter to provide a comprehensive overview of all the ways in which New Yorkers are adapting to the realities of a changing climate. Instead, this section focuses on exploring key dimensions of adaptation and resilience that can accelerate equitable responses to climate change in the state.

**TABLE 8‐4:** Examples of adaptation measures in the society and economy sector.

Topic area	Adaptation measure	Where to find more details
Populations and migration	Many New York communities with high concentrations of older adults are joining the AARP's Age‐Friendly Network as a way of preparing for more aging‐in‐place, strengthening social networks, and combating isolation—all important actions for enhancing resilience in these communities.	Section 4.1.2
Populations and migration	Many communities are taking steps to prepare for flooding and manage potential displacement.	Sections 3.1.2 and 5.4
Economy	The New York City Department of Small Business Services runs a Business Preparedness and Resiliency Program designed to help small businesses prepare for emergencies and enhance the resilience of their operations, assets, and physical spaces.	Section 5.3
Economy	To offset expected reductions in snow‐related tourism due to changing winter conditions, the Adirondack region is taking steps to position itself as a biking destination, including through the offseason use of ski facilities and other winter sports infrastructure for activities such as mountain biking and bicycle touring.	Adirondacks as a Cycling Destination case study
Education	Schools are installing air conditioning to address concerns about high heat and humidity, which can lead to mold growth and problems with indoor air quality. New York City invested more than $400 million to upgrade electrical systems and purchase air conditioners for classrooms.^156^	Sections 3.3.3 and 3.3.4
Education	Many colleges in the state have developed climate preparedness plans that include actions such as moving sensitive equipment out of flood‐prone basements, installing backup generators, adding air conditioning in dorms, and developing emergency warning systems.	Section 3.3.2
Culture, arts, and historic preservation	The Wild Center, a natural history center in Tupper Lake, New York, holds an annual Youth Climate Summit to convene and empower young people to implement climate action plans in their schools and communities.	Section 5.2
State and local government	The New York Rising Community Reconstruction Program, run by the Office of Resilient Homes and Communities, is a state‐led effort to promote equitable community‐driven resilience planning. The program is assisting 124 communities severely damaged by extreme weather events.^293^	Section 5.4

In 2019, when New York took on a national climate leadership role with the signing of the Climate Act, the state also signaled its intention to lead in other areas, such as climate justice and just transitions. Climate change presents many opportunities for state and local governments, the private sector, and civil society to identify innovative responses that contribute to equitable and just transitions to sustainability.^37^, ^38^, ^39^ This section looks at climate solutions that attempt to benefit all New Yorkers, with a special focus on those who are most vulnerable or who bear the greatest burden from climate change. Section 5.1 begins by breaking down the different types of equity that need to be considered in the pursuit of just climate solutions. Section 5.2 provides examples of work being done to prepare young New Yorkers and others for climate change and summarizes evidence that climate education can reduce climate risk vulnerability. Section 5.3 describes training programs that provide support for vulnerable workers and small business owners who are most at risk from climate‐related economic disruptions. Section 5.4 highlights some programs that are promoting climate resilience through equitable and inclusive planning processes that involve collaboration between governments, affected communities, nonprofits, and the private sector.

### 5.1 Advancing equity through climate solutions

Centering equity in adaptation and resilience actions is critical for ensuring that climate change responses are inclusive and sustainable at the local level.^256^, ^294^, ^295^, ^296^, ^297^ An equity focus for adaptation and resilience means involving local communities in all phases of adaptation planning and requires attention to long‐standing environmental justice practices as well as more recent climate justice discussions.^255^, ^294^, ^298^, ^299^

Environmental and climate justice literature commonly cites four forms of equity.^256^, ^300^, ^301^, ^302^ Awareness of the following different forms of equity is critical for developing inclusive and durable climate change responses:^256^

- **Distributional equity** is concerned with drawing attention to uneven capacities to adapt across different communities and groups, as well as to inequalities in the benefits and harms of adaptation actions.^64^, ^256^
- **Procedural equity** focuses on the representation and inclusion of affected groups and communities in adaptation processes, decision‐making, and resilience actions.^256^
- **Contextual equity** and **recognitional equity** are overlapping concepts that emphasize underlying factors including pre‐existing social and economic inequalities, historical legacies of land dispossession, and racial and ethnic discrimination and related injustices that create an “uneven playing field” for particular communities.^256^, ^300^

An examination of climate responses underway in New York State shows a number of high‐profile initiatives that are attempting to put these equity principles into action. Sections 5.3 and 5.4 provide some examples of such initiatives. Perhaps the most notable example is the Climate Act itself, which in its requirements and procedures reflects an awareness of all four types of equity. For example, the Climate Act takes on questions of distributional equity by requiring that disadvantaged communities receive at least 35% of the benefits of spending and investments under the new law.^303^, ^304^ It addresses the need for procedural equity by calling for the formation of a Climate Justice Working Group, made up of representatives from environmental justice communities across the state, to advise on the draft scoping plan and undertake a large‐scale project to identify and map the state's disadvantaged communities. The Climate Act also embraces principles of contextual/recognitional equity by acknowledging, in its language, the heightened vulnerability of disadvantaged communities that “bear environmental and socioeconomic burdens as well as legacies of racial and ethnic discrimination” and by taking concrete steps to prioritize the health and safety of those communities.^303^

In New York State, vulnerable communities have often taken the lead in calling for and defining equitable responses to climate‐related threats. For example, environmental justice organizations played a critical role in drawing attention to the connections between environmental and climate justice, as reflected in New York's Climate Act^303^ and in the White House Justice40 Initiative, which aims to direct 40% of the overall benefits of certain federal investments to disadvantaged communities.^305^

### 5.2 Educating New Yorkers about climate change

International organizations, from the United Nations to the Organisation for Economic Co‐operation and Development, have recognized the pivotal role that education plays in preparing people to address climate change.^306^, ^307^ Education enables people to understand the magnitude of the climate crisis and can provide them with the knowledge and skills to recognize and solve climate‐related challenges.^307^ As described below, there is some evidence that climate education reduces an individual's vulnerability to climate hazards.

This section reviews efforts to provide climate education in New York State, both through schools and universities and through nonformal education programs. Workforce training is covered in Section 5.3.

#### 5.2.1 Preparing young New Yorkers for climate change

Framing adaptation and resilience through a lens of equity draws attention to intergenerational equity and the fact that young people will bear a greater burden from climate change than older generations.^297^

Within New York State, a number of youth‐focused adaptation and resilience actions are helping to prepare young people for ongoing and future climate change. Schools and universities can play a key role in educating young people about climate change.^308^, ^309^, ^310^, ^311^ One study found that including climate change education as part of the core curriculum at higher education institutions may lead to a long‐term reduction in individual and collective carbon footprints.^312^ Another study produced evidence that climate education can reduce climate risk vulnerability, leading to a drop in death tolls and property damage.^313^ College and universities in New York State can contribute to climate solutions by making climate education part of their core course offerings. Climate change also offers important opportunities for curricular innovation for younger students. In 2016, the New York State Education Department adopted learning standards that introduce climate change in middle school.^314^ While lesson plan development is up to teachers, several organizations have developed resources to help. For example, the New York City Department of Environmental Protection offers learning modules and resources for teachers. The Inquiry to Student Environmental Action project has developed a carbon footprint calculator for use by students.^315^

Looking beyond the classroom, several organizations have developed climate action programs that appeal to youth and college‐aged individuals directly. For example, The Wild Center in Tupper Lake started a Youth Climate Summit in 2009. The program works globally to convene and empower young people to implement climate action plans in their schools and communities. Originally aimed at high school students, it now includes undergraduates. A New York State delegation from the Wild Center attended the 2021 United Nations Climate Change Conference in Glasgow.^316^ The Youth Climate Summit in New York also trains individuals for participation in Climate Smart Communities Councils and has equipped youth to engage with local planning boards to initiate Climate Smart Communities Task Forces and other community efforts.^317^

#### 5.2.2 Nonformal climate education programs

Nonformal education programs are also vital for providing climate education to New Yorkers of all ages. For example, the Cornell Climate Stewards program, developed by Cornell University and New York Sea Grant, trains volunteers to plan and implement climate change education, mitigation, and adaptation projects at the local level. In 2021, its inaugural year, the program provided 12 weeks of research‐based training to a cohort of more than 50 volunteers from counties across the state.^318^ Trainees who complete the program are then expected to take climate‐related action—whether by supporting the capacity of local government, holding educational sessions for other members of the public, or completing community‐based projects. Within Monroe County, volunteers from the inaugural year have helped to start community gardens in neighborhoods that are food deserts, provided workforce development training for clean energy installations, and launched campaigns for “healthy lawns” and pollinator protection.^319^ As noted in Section 5.4, ensuring that nonformal programs of this type are widely accessible to LMI New Yorkers, particularly racial and ethnic minorities and immigrant communities, often requires partnerships between local governments and community‐based nonprofits.

Museums and other cultural institutions also play a key role in educating the public about climate change. According to research published by the American Alliance of Museums in 2021, “the public continues to regard museums as highly trustworthy—ranking second only to friends and family, and significantly more trustworthy than researchers and scientists, NGOs [nongovernmental organizations] generally, various news organizations, the government, corporations and business, and social media.”^320^ Because of their standing in their communities, which extends across the political spectrum, museums can play a major role in climate change education. Science museums are natural venues for such education. For example, The Wild Center in Tupper Lake opened a climate change exhibit that could serve as a model for other museums that want to incorporate local and Indigenous knowledges into their exhibitions and emphasize climate change and its intersections with nature, history, and the arts.^316^ Other types of museums can also present different perspectives on the issue, hewing to their mission as well as appealing to audiences who do not consider themselves “science types.” One recent example was the Princeton University Art Museum's 2018−2019 exhibit “Nature's Nation: American Art and Environment” and its accompanying catalog, which traced environmental awareness in American art over the last six centuries.^321^

### 5.3 Supporting workers and small business owners through education and training

As described in Section 4.4.2, many workers face special vulnerabilities to climate‐related shocks to the economy. For example, hourly wage workers in retail, hotels, restaurants, and related sectors may experience loss of jobs or income from shutdowns related to major storm events. Workforce training programs can help workers transition from vulnerable occupations into more stable positions. Training can also prepare workers, especially LMI individuals, to take advantage of job opportunities in the disaster recovery and resilience fields. Recognizing that one of the benefits of adaptation and resilience actions is that they create jobs, these types of programs address issues of distributional equity by connecting individuals with jobs in areas that have a history of high poverty and unemployment rates.

At the state level, the Office of Resilient Homes and Communities (formerly the Governor's Office of Storm Recovery) has funded several such workforce development programs in collaboration with nonprofit partners. For example:

- The Southwest Brooklyn Industrial Development Corporation runs a workforce training program for LMI individuals in the Red Hook community of Brooklyn. The program helps trainees develop work skills needed to take advantage of employment opportunities in the emerging resilience construction field.
- The Disaster Recovery Workforce Training Program targets low‐income and very‐low‐income individuals and public housing residents from certain neighborhoods in Brooklyn and Staten Island.^322^ Run by Rebuilding Together NYC with funding from the Office of Resilient Homes and Communities, the program includes a 6‐week course that gives participants technical and practical experience in the construction and demolition trades, with the potential to receive certifications from the Occupational Safety and Health Administration and the Home Builders Institute.
- The New York State Extreme Heat Action Planning process is an interagency effort to address the impacts of extreme heat and develop equity‐grounded strategies to manage those impacts. A critical component of the plan is regular stakeholder feedback and input from communities across the state to ensure the Extreme Heat Action Plan is as representative as possible and reduces the impacts faced by heat‐vulnerable communities.^323^

In developing these types of workforce training programs, it is essential that government agencies partner with community‐based nonprofit organizations to ensure frontline communities can access and participate in them, particularly racial and ethnic minorities and immigrant communities with limited English proficiency. Online resources are often inaccessible to immigrant and low‐income communities with limited digital literacy and broadband access. Partnering with immigrant‐serving community organizations helps ensure that immigrant and LMI New Yorkers, including residents, homeowners, and frontline workers, are able to participate in efforts to prepare for climate change.

Small business owners are another vulnerable group, partly because they tend to have limited resources for developing and implementing climate preparedness plans. After a disaster event, they may face greater difficulty in obtaining rebuilding loans and may be less likely to reopen than larger businesses. The New York City Department of Small Business Services’ Business Preparedness and Resiliency Program is designed to help small businesses prepare for emergencies and enhance the resilience of their operations, assets, and physical spaces.^324^ The program provides resilience workshops, onsite resilience assessments, and post‐assessment grants. The program's online resources educate businesses on best practices to reduce risk and connect them to resilience services. By 2018, 3 years after the program's founding, almost 1000 businesses had taken part, with support from both the Department of Small Business Services and the New York State Governor's Office of Storm Recovery.^325^

### 5.4 Promoting climate resilience through collaborative planning

Promoting climate resilience through equitable and inclusive processes requires collaborative partnerships between civil society; the private sector; and federal, state, and local governments.^182^ These efforts are most effective when they follow principles of procedural equity, meaning that local community representatives should be at the table in all phases of adaptation and resilience planning, and community voices and perspectives should be prioritized.^256^, ^326^ The paragraphs below provide examples of the kinds of collaborations that are taking place in New York State.

#### 5.4.1 State‐led initiatives

State and local governments have the power to lead and contribute to resilience efforts in meaningful ways that extend beyond the scope of the federal government. Preparing for community resilience can be part of a comprehensive planning process undertaken by state and local governments that includes climate adaptation and mitigation, hazard mitigation, and post‐disaster recovery. Government agencies should conduct this planning in collaboration with community‐based organizations, which are best equipped to identify local vulnerabilities, persistent climate risks, and vulnerable population groups and occupations.

New York, perhaps more than most other states in the country, has an extensive network of community‐based organizations actively involved in the development of comprehensive climate action plans. Organizations like Cornell Cooperative Extension work with these community organizations, as well as with local governments, to provide technical assistance. Cornell Cooperative Extension also serves as an intermediary between community‐based organizations and the state government, delivering climate education programs and promoting the implementation of specific actions to bolster long‐term resilience and responsive capacity.

New York's state government runs a number of programs that promote collaborative planning on climate issues. Examples include the following:

- The CLEAR (Coastal Lakeshore Economy and Resiliency) program is an example of a collaborative alliance between government, community stakeholders, and the private sector that addresses questions of procedural equity. Through the program, state experts and professional planning firms work directly with affected community members to develop comprehensive and long‐term coastal resilience plans and strategies in flood‐prone regions along Lake Ontario, the upper St. Lawrence River, and the lower Niagara River.
- The New York Rising Community Reconstruction Program, run by the Office of Resilient Homes and Communities, provides another example of a state‐led effort to promote inclusive and equitable adaptation planning. The program is a participatory recovery and resilience initiative that is assisting 124 New York State communities severely damaged by Superstorm Sandy, Hurricane Irene, and Tropical Storm Lee. It includes more than 300 projects undertaken in partnership with local governments, nonprofit partners, and the Dormitory Authority of the State of New York.^280^
- The Climate Smart Communities program, established in 2009, supports local governments in leading their communities to reduce greenhouse gas emissions and adapt to the effects of climate change. Jointly sponsored by seven New York State agencies, the program offers technical assistance and a comprehensive list of suggested actions to registered communities willing to make a formal commitment to act by adopting a pledge.^327^ As of July 2022, there were nearly 9.5 million people living in 381 certified Climate Smart Communities across the state. These communities are eligible to receive Climate Smart Communities grants, as well as rebates for zero‐emission vehicles and infrastructure.^327^
- In 2015, the Governor's Office of Storm Recovery (now the Office of Resilient Homes and Communities) teamed with the nonprofit Center for NYC Neighborhoods to launch the Residential Technical Assistance Pilot Program, an effort to provide seven storm‐impacted communities in New York City with technical assistance on residential property resilience. The program's aim was to facilitate the completion of residential resilience audits, offer retrofitting advice, and provide financial counseling for qualified homeowners, primarily LMI individuals.^328^ In a related effort, the Center for NYC Neighborhoods developed the FloodHelpNY online platform, which informs homeowners in New York City about their flood risks, what they can do to protect their homes, and how they can protect their finances from flood damages.^329^

#### 5.4.2 Community‐led initiatives

Local communities are also initiating collaborative efforts to identify climate solutions. Examples include the following:

- The Climate Solutions Accelerator of the Finger Lakes region's Color Your Community Green program provides a platform for concerned individuals (organized by town) to come together to identify locally relevant climate solutions and determine the best path forward for their community.^330^
- New York City−based organizations such as WE ACT and UPROSE have developed community‐centered climate change response strategies that mobilize local community participation in emergency preparedness and support just transitions through community solar energy development, wind turbine assembly, and related measures.^331^, ^332^
- In Buffalo, the community‐based organization PUSH Buffalo has mobilized residents with a focus on housing discrimination, jobs, and environmental justice.^333^

Similarly, Tribal Nations throughout the United States have developed climate change adaptation plans that reflect the needs and concerns of their communities.^334^ Within New York State, both the Saint Regis Mohawk Tribe and Shinnecock Nation have developed adaptation plans.^251^, ^335^ Others, including the Seneca and Oneida Nations, are in the process of developing strategic climate and energy plans.^336^ These plans draw on Indigenous knowledge to identify proactive strategies that respond to climate change pressures on the valued ecological resources and cultural traditions of each Tribe or Nation.

## 6 LOOKING AHEAD

This section looks at opportunities for positive change that can grow out of climate adaptation efforts and identifies emerging topics and research needs in the society and economy sector. The section concludes by summarizing the major findings and recommendations presented in the chapter.

### 6.1 Opportunities for positive change

The work of adapting to a changing climate and advancing community resilience can present opportunities and cobenefits. Indeed, many of the adaptation strategies described in Section 5 offer wide‐ranging benefits. For example, training programs that prepare workers to join the growing resilience construction field have created new pathways to employment in communities with high poverty rates and limited job opportunities. The paragraphs below describe some other beneficial opportunities that businesses, governments, and other institutions can access by taking action to address climate‐related challenges.

#### 6.1.1 Social and economic opportunities

- **Reversing population declines**. Climate‐related population displacement and migration from parts of the United States that have become hotter, dryer, and more prone to wildfires can provide an opportunity for much‐needed population gain in regions of New York State that have experienced population declines over recent decades. Cities such as Buffalo, Syracuse, and Utica may be able to attract new migrants to areas that have lost population. Development or remodeling of housing and retrofitting and improvement of infrastructure may provide new job opportunities in construction and related sectors.
- **Adapting supply chains**. As the COVID‐19 pandemic demonstrated, global supply chains can be severely disrupted by exogenous forces. Manufacturers and other New York businesses that rely on global supply chains of imported parts are vulnerable to climate events that cause shutdowns in overseas plants and disrupt international shipping. Due to New York's economic strength, the state has an opportunity to be a leader in adapting supply chains to make them more resilient in the face of climate‐related threats. The state's Green CHIPS program, which offers tax incentives for companies that undertake environmentally friendly semiconductor manufacturing and supply chain projects in New York, is an example of the kind of initiative that can localize supply chains and reduce dependence on less climate‐resilient countries.^337^ As part of the program, Micron Technology announced in October 2022 that it will invest $100 billion over the next 20 years to build a semiconductor manufacturing campus in Onondaga County, bringing thousands of high‐paying jobs to Central New York and creating a domestic supply chain of “Made in New York” microchips for use in products from electric vehicles to personal computers and smartphones.
- **Creating jobs in a green economy**. The work of reducing greenhouse gas emissions and adapting to changing climate conditions creates opportunities for new businesses and jobs. Specific economic opportunities have emerged in energy efficiency, electrification, manufacturing, and renewable energy, with related opportunities for local suppliers. Workers transitioning into these fields from more vulnerable occupations will help increase the state's economic security and resilience. As discussed in the Agriculture and Energy chapters of this assessment, climate change also presents opportunities for job creation in areas such as sustainable agriculture and renewable energy.

#### 6.1.2 Educational and training opportunities

- **Creating regionally integrated training and apprenticeship programs**. New job creation opens the door for workforce development organizations to cocreate regionally integrated training and apprenticeship programs, addressing local needs and providing an alternative to vulnerable occupations. There are opportunities to develop comprehensive training curricula, micro‐credentialing courses, and certification programs aimed at offering new career pathways and opportunities to reduce unemployment and underemployment among communities of color, undocumented immigrants, formerly incarcerated individuals, and people from violent backgrounds. There are also opportunities for cocreating training curriculums in collaboration with Indigenous Peoples.

#### 6.1.3 Opportunities for museums, arts organizations, and historic organizations

- **Using state and regional networks to foster collaboration and capacity building**. Many of New York's historic and arts organizations rely on state and regional networks for information sharing, training, and grant funding. The Museum Association of New York and the New York State Council for the Arts are particularly important to smaller institutions, which often have few professional staff members or resources. There is an opportunity for these organizations to use their training capacity, networks, and funding powers to play a larger role in helping small cultural institutions statewide prepare for and adapt to climate change through information sharing and collaboration. Models for this work are not hard to find. The national 2030 Districts Network, for example, has a museum group that has regular meetings on sustainability and climate change issues for museums.^338^ The New England Museum Association and federal agencies like the National Park Service have developed publicly available strategies for assessing and preparing for risks.

#### 6.1.4 Opportunities for local and state governments

- **Addressing equity concerns in adapting affordable housing for climate change**. Local governments have an opportunity to incorporate equity, racial justice, and environmental justice into their climate action, resilience, and hazard mitigation plans. This can be done in partnership with organizations like Cornell Cooperative Extension and New York Sea Grant, as well as with community‐based organizations. For example, partnerships between local governments and nonprofit affordable housing organizations can help build these organizations’ capacity and resources to implement adaptation measures in the affordable housing stock they manage for low‐income residents. Adaptation to climate change in the context of housing shortages also offers local governments an opportunity to work with community‐based and nonprofit organizations to create land trusts to support long‐term housing affordability and to bridge the gap between rental housing and homeownership.^339^ The Buildings chapter offers many examples of how people can adapt buildings to climate change and what state programs promote and fund these changes.
- **Using public‐private‐philanthropic partnerships to develop equitable climate adaptation programs at the local scale**. By partnering with local nonprofit and community‐based organizations, including urban development agencies and affordable housing organizations, local governments can build capacity to identify and implement equitable climate adaptation programs. Through the development of new types of public‐private‐philanthropic partnerships, local governments may be able to use innovative financing to fund energy efficiency and weatherization projects, as well as the electrification of the local building stock and transportation systems. These partnerships also allow for the designation of cold weather shelters and cooling centers, as well as for the development of community‐driven emergency response networks. They offer an opportunity to make progress toward community‐wide financial inclusions, enabling low‐income, undocumented, formerly incarcerated, and immigrant populations to have affordable access to financial products and services, including insurance and second‐hand electric vehicle loans.

#### 6.1.5 Opportunities for financial institutions

- **Updating methodologies for financial risk assessment**. Climate stress tests have been developed in the academic literature to translate climate scenarios into adjustments in the financial valuation of securities and contracts, and into investors’ risk metrics and financial losses.^340^ Similarly, actuarial models to determine insurance reserves in the face of climate risk uncertainty can be adjusted.

### 6.2 Emerging topics and research needs

Many issues emerged during the development of the chapter that could benefit from further investigation. In terms of next steps for research and policy development, priorities include the following:

- Addressing intersectional climate change vulnerabilities affecting LMI populations, particularly historically marginalized groups such as Indigenous Peoples, environmental justice communities, and immigrant populations.
- Investigating exposures and the adaptive capacity of middle‐income populations who may not perceive themselves as vulnerable until after an extreme event has occurred.
- Identifying impacts of climate change on housing throughout the state, especially in light of the ongoing affordable housing shortages and planning needs associated with changing patterns of migration and population settlement.
- Understanding the role of financial regulators in climate‐related financial disclosures and climate financial risk assessments, including understanding the definition of climate scenarios, climate stress tests, and financial valuation.
- Identifying potential unintended consequences of resilience actions, such as the possibility of climate gentrification in urban areas or the possibility of damage to sensitive ecological areas as a result of higher levels of tourism during “shoulder” seasons.
- Preparing for supply chain disruptions associated with future extreme climate events, including compound events that affect more than one region.
- Mapping and assessing transmission channels through which climate shocks that affect specific economic sectors may be amplified or transmitted to other regions.
- Developing strategies to protect vulnerable workers, foster resilience in small businesses, and support just transitions in rural, resource‐dependent economies.
- Supporting climate change education and workforce training, including the development of new K−12, university, and adult educational curricula.
- Promoting preparedness and resilience for local governments, schools, and arts and cultural institutions, including through the development of mechanisms for collaboration among small governments, the private sector, and civil society.

Attention to these issues will be critical as the state develops strategies and plans to both adapt to and thrive under a changing climate.

### 6.3 Conclusions

Climate change has already had significant impacts on society and the economy in New York State, and more impacts lie ahead. These impacts overlap and interact with other social, economic, and health stressors affecting the state, including the aging of the population, declining population in many rural areas, high levels of wage and income inequality, supply chain disruptions, high rates of inflation, and other lingering effects of the COVID‐19 pandemic. As described in other chapters of this assessment, the impacts of climate change are also exacerbated by other environmental stressors affecting the state, such as development pressures on forests and agricultural lands, the spread of invasive species, and water supply and quality concerns. This chapter investigated the effects of climate change in five major subareas: impacts on populations and migration, economic impacts, educational impacts, cultural impacts, and impacts on local and state government. The chapter also identified key vulnerable populations, groups, and regions and opportunities for advancing adaptation and resilience.

Concerning the impacts of climate change on populations and migration, the chapter emphasized the critical need for attention to aging populations in the state, particularly in rural areas, as well as a need to plan for population movement, including shifts of population out of vulnerable, flood‐prone coastal areas and a possible influx of climate migrants into cities such as Buffalo, Utica, and Syracuse, where populations are currently stable or shrinking. The chapter also identified high levels of exposure to climate risks among populations living on the Atlantic coast, where sea level rise and coastal flooding are growing threats. Other areas that are subject to growing climate threats include flood‐prone inland regions in both urban and rural areas, environmental justice communities, Indigenous communities, and communities located along the Great Lakes.

Regarding economic impacts, the chapter concluded that climate change is affecting or will affect nearly every economic sector in the state and that these impacts are particularly acute in rural regions that are economically dependent on agriculture, natural resources, and outdoor tourism. The chapter highlighted the impacts of climate change on the insurance and financial sectors, where the exposure of economic assets such as real estate, manufacturing facilities, and infrastructure is creating significant new risks. The chapter noted the disproportionate impacts of climate extremes on outdoor workers; frontline service workers; and public servants such as police, firefighters, and emergency responders. The chapter also highlighted disproportionate impacts on small businesses, especially immigrant‐owned businesses.

In the education sector, substantial investments may be needed to ensure that schools can remain open and educational delivery can continue amidst rising numbers of extreme events, including heat waves, heavy rainfall events, and winter storms. Investment in educator training and preparation of the workforce for climate change will be important objectives for secondary and post‐secondary schools. Within the culture, arts, and historic preservation sector, small museums and cultural institutions are particularly at risk due to limited resources and adaptive capacity. Climate change will also pose significant challenges for local governments, especially in the state's resource‐strapped and fiscally constrained smaller cities and rural communities. Ensuring that these municipalities have the resources and capacity to adapt is a critical challenge for New York State.

While climate change is affecting the life of every resident of New York State, the impacts are most immediate for populations and communities that are already vulnerable or have limited capacity to cope with social, economic, and environmental stressors. Many factors influencing vulnerability are intersectional, such that particular groups often face multiple forms of disadvantage due to factors such as income status, race or ethnicity, age, gender, or sexual identity. Populations likely to experience these intersectional vulnerabilities include LMI individuals, older adults, the very young, the unhoused, members of the LGBTQ+ community, Tribal communities and Indigenous Peoples, historically marginalized racial and ethnic minorities, and new immigrant groups.

Finally, the chapter highlighted several critical topics for advancing adaptation and resilience. These include the need to center equity in adaptation planning; to facilitate education about climate change for all New Yorkers; and to foster collaboration among public institutions, the private sector, nonprofits, and civil society in the development of effective climate responses. As New York prepares for the continued impacts of climate change, attention to these topics will help to ensure that the state's responses are equitable, sustainable, and forward‐looking.

## 7 TRACEABLE ACCOUNTS

Traceable accounts examine each key finding in depth. They provide citations that support each assertion and present the authors’ assessment of confidence in each finding.

### 7.1 Key Finding 1

**Climate change is already affecting and will increasingly affect nearly every dimension of New York State's economy**. Climate hazards such as rising temperatures and more extreme weather events will directly or indirectly affect nearly all economic sectors in the state, from natural resource−based industries to manufacturing, retail, and financial services. To benefit all New Yorkers, climate solutions will need to consider those who are disproportionately burdened by economic disruption, such as small businesses; fiscally constrained, small, and rural municipalities and cultural institutions; frontline workers; and essential public servants including police, firefighters, and teachers.

#### 7.1.1 Description of evidence base

A large body of studies have documented the economic impacts of climate change in the United States, the Northeast, and New York State.^50^, ^64^, ^65^ The evidence that small businesses are especially vulnerable comes from multiple sources that document these vulnerabilities in New York and elsewhere.^277^, ^284^, ^341^ The evidence that frontline workers are disproportionately vulnerable comes from Berube and Bateman^287^ and other literature documenting differential vulnerabilities to COVID‐19 among workers in New York, as well as from news reports and studies of the economic impacts of Superstorm Sandy.^64^, ^288^ The impacts of climate change on worker health are documented in other chapters of the assessment.^62^, ^153^

A number of studies document the impacts of climate change on schools, learning, and educational delivery,^139^, ^142^, ^150^ as well as the impacts on local governments,^191^ including impacts in New York State.^184^ Additional evidence comes from the personal and professional knowledge and experience of the Technical Workgroup and Sector Advisors, particularly regarding impacts on museums and arts institutions in the state.

#### 7.1.2 New information and remaining uncertainties

While there is strong evidence that climate change will impact the economy of New York State, there are remaining uncertainties about the timing and total costs of these impacts for the economy as a whole and for each major sector as well as the ripple effects of impacts on other sectors. Analysis of this type is currently underway in a separate study of the economic impacts of climate change in the state. The indirect impacts of climate change on income levels, employment, and labor productivity are also uncertain.

Given the small body of New York State−focused studies on these topics, many uncertainties remain about the full extent of climate change impacts on the arts and culture, education, and government sectors.

#### 7.1.3 Assessment of confidence based on evidence

Given the evidence base, there is **very high** confidence that climate change will have increasing impacts on New York State's economy, and **high** confidence that small businesses and frontline workers are especially vulnerable. Given the limited evidence, particularly regarding impacts in New York State, there is **medium** confidence in the finding that climate change will have significant impacts on local government, schools and educational delivery, and arts institutions, and **medium** confidence in the locational patterns of vulnerability.

### 7.2 Key Finding 2

**New York State's older residents are more vulnerable and less able to adapt to heat, flooding, and other climate hazards**. New York State's population is aging, especially in rural areas where the portion of the population over 65 is growing at a much faster rate than in urban areas. Addressing the unique vulnerabilities of older New Yorkers will require special attention to emergency preparedness and disaster relief planning.

#### 7.2.1 Description of evidence base

Demographic data from the U.S. Census Bureau provide evidence of the current population age structure in New York State.^342^ Population projections come from the Cornell Program on Applied Demographics and are based on the American Community Survey and other census data inputs.^61^ Evidence that older individuals are more vulnerable and less able to adapt comes from a large body of empirical studies of climate change and health outcomes,^210^, ^212^ as well as from studies and reports documenting New York State's and New York City's experiences during Superstorm Sandy and other extreme events.^211^, ^212^ Evidence that communities with higher concentrations of older residents face added adaptation challenges comes from the personal and professional knowledge and experience of the Technical Workgroup members and Sector Advisors.

#### 7.2.2 New information and remaining uncertainties

Remaining uncertainties include the magnitude and nature of challenges faced by communities with large proportions of older individuals. While older populations face greater risks, what these risks will mean for different New York communities and the precise capacities of communities to respond to these added risks have not been assessed.

#### 7.2.3 Assessment of confidence based on evidence

Given the evidence base, there is **very high** confidence that New York State's population is aging, especially in rural areas, and that it is older than many other parts of the country. There is also **very high** confidence that older populations are especially vulnerable to climate stresses. There is **medium** confidence that communities with large proportions of older residents will face added adaptation and resilience challenges.

### 7.3 Key Finding 3

**Climate change vulnerabilities intersect with and are exacerbated by underlying and systemic social/economic stressors and fragilities**. Low‐income populations in New York State, notably those who are Indigenous, people of color, immigrants, unhoused, or live in rural areas, are more vulnerable due to existing forms of disadvantage and marginalization, including legacies of displacement, racial and ethnic discrimination, limited access to resources, and higher exposure to environmental pollutants. Recognition of and attention to the needs of historically underserved and overburdened populations are critical elements of equitable and effective resilience planning.

#### 7.3.1 Description of evidence base

These findings are based on a large body of research literature on vulnerability, the root causes of vulnerability, and the linkages between climate vulnerability and other social and economic stresses.^30^, ^198^, ^199^ Within this literature, a growing body of studies demonstrate the racialized nature of vulnerability, as well as the heightened vulnerabilities of Indigenous Peoples and environmental justice communities.^200^, ^201^, ^202^, ^256^ The evidence base for vulnerabilities of other socially marginalized populations comes from the personal and professional knowledge and experience of the Technical Workgroup and Sector Advisors.

#### 7.3.2 New information and remaining uncertainties

Because the impacts on some marginalized populations (e.g., formerly incarcerated individuals, LGBTQ+ individuals) have not been studied in detail, uncertainties remain about the nature and magnitude of the vulnerabilities faced by these populations, as well as the best strategies to reduce those vulnerabilities. There are also uncertainties about effective strategies to address root causes of vulnerability and support the resilience of underserved and marginalized communities.

#### 7.3.3 Assessment of confidence based on evidence

Given the strong evidence base supporting the determinants of vulnerability, there is **very high** confidence that vulnerabilities to climate change intersect with other social stresses and fragilities. Given the smaller (but still robust) body of evidence about the racialized nature of vulnerability and the heightened vulnerability of Indigenous Peoples and environmental justice communities, there is **high** confidence in this finding. Due to the more limited body of evidence about vulnerabilities of marginalized populations other than Indigenous Peoples and environmental justice communities, there is **medium** confidence that they are more vulnerable to climate change impacts.

### 7.4 Key Finding 4

**Climate change threatens Indigenous communities in New York State and their natural resource**−**based cultural heritages and traditions**. Loss of heritage sites due to sea level rise, reductions in opportunities for traditional livelihood practices such as ice fishing, and loss of natural resources that are central to cultural practices are climate‐driven impacts threatening Indigenous Peoples living in the state. Indigenous knowledges and cultural traditions are important sources of information about natural hazard risks, climate change exposures, and natural resource management practices, especially when collaboratively incorporated into adaptation and resilience responses.

#### 7.4.1 Description of evidence base

The evidence base demonstrating the impacts of climate change on Indigenous Peoples comes from published reports and scholarly literature^50^, ^249^ and from the personal and professional knowledge and experience of the Technical Workgroup, Sector Advisors, and Indigenous Working Group. The evidence base supporting the value of local knowledge comes from a growing body of literature on the importance of local and Indigenous knowledges in adaptation planning^50^, ^202^, ^256^ and from the knowledge and experience of the Technical Workgroup, Sector Advisors, and Indigenous Working Group.

#### 7.4.2 New information and remaining uncertainties

Given the small body of New York State−focused studies on these topics, many uncertainties remain about the full extent of climate change impacts on cultural heritage and Indigenous communities.

#### 7.4.3 Assessment of confidence based on evidence

Given the strong evidence base supporting the cultural impacts of climate change, there is **high** confidence in the finding that climate change will have significant impacts on Indigenous communities and their cultural heritage and traditions.

### 7.5 Key Finding 5

**Innovative responses to climate change present opportunities to contribute to equitable and just transitions to sustainability**. Each sector and every community in New York State has the potential to contribute to climate solutions that reduce vulnerabilities, foster resilience, and enhance equity. Local and state governments, schools and universities, nonprofits, museums and cultural institutions, and the private sector all play vital roles in raising climate change awareness, supporting educational and workforce training efforts, and identifying opportunities for innovation that will be necessary to prepare the state for a changing climate. Centering equity in adaptation and resilience actions and aligning these actions with greenhouse gas mitigation strategies is critical for a successful and sustainable climate change response.

#### 7.5.1 Description of evidence base

The Technical Workgroup and Sector Advisors suggested numerous examples of opportunities to enhance equity and sustainability while responding to climate change. Their suggestions form part of the evidence base for these findings. Evidence also comes from a growing body of scholarly and gray literature reports on climate justice and just transitions to sustainability.^37^, ^38^, ^39^ The evidence base for centering equity comes from a growing body of literature showing the need for attention to equity in adaptation planning.^256^, ^294^, ^295^, ^296^, ^297^, ^301^

#### 7.5.2 New information and remaining uncertainties

Equity in climate change responses is a rapidly growing field. Uncertainties remain on best practices for incorporating equity into adaptation and mitigation policies, plans, and actions. Understanding and acceptance of the role and value of local knowledge in adaptation planning is uneven within the scientific, research, and policy communities. There are many uncertainties about how to identify and foster equitable and sustainable climate responses in New York State. There are also uncertainties about how to create incentives for communities and businesses to take advantage of opportunities associated with climate change.

#### 7.5.3 Assessment of confidence based on evidence

There is **high** confidence in this finding based on (1) the strong evidence base about the importance of equity in successful adaptation and mitigation planning, (2) the growing evidence supporting the value of local knowledge, (3) the growing body of research and gray literature on the alignment of adaptation and mitigation, and (4) the expertise of the Technical Workgroup and Sector Advisors.

## AUTHOR CONTRIBUTIONS

L.A.‐T.: Conceptualization of the chapter; drafting, revising, editing the manuscript; manuscript compilation and review. R.L.: Conceptualization of the chapter; drafting, revising, editing the manuscript; manuscript compilation and review. M.A.: Drafting, revising, and editing sections related to vulnerable regions, especially the Great Lakes. D.B.: Drafting, revising, and editing sections related to population, demographics, vulnerability, and adaptation. H.E.B.: Drafting, revising, and editing sections related to culture and historic preservation. R.E.B.: Drafting, revising, and editing sections relate to governance. D.B.: Drafting, revising, and editing sections related to governance, vulnerable populations, and adaptation. C.J.: Drafting, revising, and editing sections related to finance. F.P.: Drafting and reviewing sections related to environmental justice. K.S.: Drafting, revising, and editing sections related to economic impacts and adaptation.

## COMPETING INTERESTS

The authors declare no competing interests.

8

### PEER REVIEW

The peer review history for this article is available at: https://publons.com/publon/10.1111/nyas.15199.
